# An Open-Source Wireless Sensor Node Platform with Active Node-Level Reliability for Monitoring Applications

**DOI:** 10.3390/s21227613

**Published:** 2021-11-16

**Authors:** Dominik Widhalm, Karl M. Goeschka, Wolfgang Kastner

**Affiliations:** 1 Department Electronic Engineering, University of Applied Sciences Technikum Wien, 1200 Vienna, Austria; karl.goeschka@technikum-wien.at; 2 Automation Systems Group, Faculty of Informatics, TU Wien, 1040 Vienna, Austria; wolfgang.kastner@tuwien.ac.at

**Keywords:** sensor node, fault diagnosis, node-level, energy-efficiency, wireless sensor network

## Abstract

In wireless sensor networks, the quality of the provided data is influenced by the properties of the sensor nodes. Often deployed in large numbers, they usually consist of low-cost components where failures are the norm, even more so in harsh outdoor environments. Current fault detection techniques, however, consider the sensor data alone and neglect vital information from the nodes’ hard- and software. As a consequence, they can not distinguish between rare data anomalies caused by proper events in the sensed data on one side and fault-induced data distortion on the other side. In this paper, we contribute with a novel, open-source sensor node platform for monitoring applications such as environmental monitoring. For long battery life, it comprises mainly low-power components. In contrast to other sensor nodes, our platform provides self-diagnostic measures to enable active node-level reliability. The entire sensor node platform including the hardware and software components has been implemented and is publicly available and free to use for everyone. Based on an extensive and long-running practical experiment setup, we show that the detectability of node faults is improved and the distinction between rare but proper events and fault-induced data distortion is indeed possible. We also show that these measures have a negligible overhead on the node’s energy efficiency and hardware costs. This improves the overall reliability of wireless sensor networks with both, long battery life and high-quality data.

## 1. Introduction

In the last two decades, wireless sensor networks (WSNs) have become an important source of information for a plethora of data services such as process automation, precision agriculture, or research (for example in biology or meteorology). These data services, however, heavily depend on the availability and quality of the input data. It has to be ensured that the data reported by a WSN are accurate. Otherwise, subsequent data services can be significantly impaired leading to wrong information and, as a result, incorrect decisions or (counter-)actions may occur (cf. [1]).

WSNs are deployed in an area of interest and are capable of measuring relevant physical quantities close to their source and, thus, can provide data with a high level of detail. Most WSN deployments can be categorized in one of two main applications depending on whether they provide continuous sensing (e.g., environmental or process monitoring) or perform event detection (e.g., forest fire detection or surveillance). While both share some common characteristics (such as the network structure), there are differences in their respective requirements especially concerning the expected lifetimes, the communication patterns, and the amount of data to be transferred. These differences have an impact on the possibility to detect faults on the node level, for example, continuous sensing provides the possibility to consider the difference between two consecutive measurements. Nevertheless, all WSNs deployments consist of spatially distributed sensor nodes realized by low-power embedded systems that measure certain physical quantities via attached sensors. Depending on the application requirements, these sensor nodes are interlinked by near-field communication (e.g., Zigbee, Bluetooth low energy (BLE)) or low-power wide-area networks (LPWANs) such as long-range wide-area network (LoRaWAN) or narrowband Internet of Things (NB-IoT).

**Figure 1:**
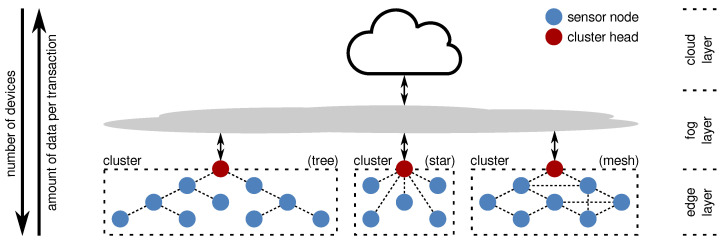
Architectural example of a clustered wireless sensor network.

In this context, sensor nodes that are located geographically close to each other are often grouped into clusters. Each cluster usually has one dedicated node (often denoted as cluster head or cluster leader) responsible for collecting the information from its neighboring sensor nodes and collectively forwarding the data to one or more central services for further processing. These cluster heads are often equipped with higher resources in comparison to the sensor nodes. An architectural example of a clustered WSN is shown in Figure 1. In the figure, the three clusters depict the three most common network topologies used in WSNs, namely tree, star, and mesh networks.

From a global point of view, the WSN operates at the boundary of the data-processing chain (at the *edge*) and, thus, the sensor nodes are sometimes denoted as *edge devices* and the cluster heads are called *edge gateways* that, together, form the *edge layer*. The data collected by the cluster heads are then either directly forwarded to a cloud system (i.e., central data endpoint) or preprocessed by intermediate systems before being uploaded to the cloud. In the latter case, the intermediate systems are commonly referred to as *fog devices* as they operate between the edge and the cloud. As shown in Figure 1, the number of devices per layer decreases from the edge to the cloud layer, while the amount of data transmitted per transaction significantly increases.

Devices in the fog and cloud layers are usually operated in a controlled environment (e.g., data centers), have a wired power supply, and are commonly equipped with vast resources regarding their processing and memory capabilities. The devices of the edge layer, however, most often operate outdoors under uncontrollable and mostly unpredictable conditions. While the cluster heads are sometimes powered by a wired power supply and have moderate resources, the sensor nodes are normally powered by batteries (with or without the possibility for energy harvesting) and have strictly limited resources. Additionally, several WSN applications densely deploy the sensor nodes to cover a wide area and/or have a fine spatial granularity resulting in large numbers of devices (ranging from tens up to thousands). This usually requires the sensor nodes to mainly consist of low-cost components to keep the deployment costs as low as possible.

### 1.1. Faults Pose a Serious Threat

Sensor nodes need to operate in a reliable and energy-efficient way to ensure accurate data acquisition while operating unattended and for long times. This poses significant challenges to the sensor node design as the combination of:

- low-cost components,
- limited resources (especially energy), and
- the often harsh environmental conditions.

Make the sensor nodes susceptible to impaired operation. Additionally, the strictly limited resources prevent the use of established reliability concepts such as redundancy by duplication or replication on the node level. As a consequence, faults occurring on sensor nodes are often the norm rather than the exception and, thus, the data reported by sensor nodes can become unreliable and inaccurate [2]. Some of these faults cause the sensor nodes to fail to operate entirely or make them unable to communicate (so-called *hard faults*). These faults can usually be easily detected by other network participants (e.g., absence of messages). More dangerous for the data quality are *soft faults* that alter the runtime behavior or data of the nodes in such a way, that the node continues to communicate but may report corrupted data. These faults not only pose a more severe threat to the WSN’s reliability, they are also much harder to detect and deal with. However, detecting hard-, but especially soft-faulty sensor nodes is crucial to ensure a reliable service (i.e., data acquisition). Therefore, measures need to be applied that allow the WSN to cope with failures of the underlying components to mitigate the risk of transmitting corrupted data (cf. [3]).

Most sensor nodes are battery-powered and, thus, have a strictly limited energy budget that prevents them from using complicated hardware circuitry or complex software algorithms to identify faults. To date, most fault detection approaches tailored for sensor nodes either rely on (cf. [4]):

1. an outlier detection in the sensor data,
2. a dense deployment to identify deviations between the measurements reported by neighboring nodes, or
3. simple node-level diagnostics such as a monitoring of the battery voltage.

Fault detection approaches based on outlier detection are often incapable of distinguishing between correct events in the measured phenomena and fault-induced deviations. The detection by incorporating neighborhood data usually requires a lot of communication between the nodes resulting in a considerable overhead on the energy consumption. Node-level diagnostics, however, rely on simplistic checks (e.g., of the battery voltage) or require specific hardware for the detection to work.

### 1.2. Active Node-Level Reliability

Based on the concept of fault indicators proposed in [4], we developed a new type of sensor node named *AVR-based Sensor Node with Xbee radio*, or short *ASN(x)*, that is completely open-source and free to use (published under the MIT license at https://github.com/DoWiD-wsn/avr-based_sensor_node).

As the name implies, the ASN(x) is based on an Atmel AVR microcontroller unit (MCU) that commonly employ a modified 8-bit Harvard reduced instruction set computer (RISC) architecture. It enables node-level fault detection by using specialized self-tests and diagnostic data, a concept we named *active node-level reliability*. The detection bases on the use of so-called fault indicators that allow us to infer information on the state of health of the sensor node’s operation and, in turn, can indicate possibly erroneous operational conditions. This additional information can be used to augment existing WSN-specific fault detection approaches (centralized as well as distributed) to improve the fault detection rate and, most importantly, to enable the distinction between data anomalies caused by rare events and fault-induced data corruption. Thereby, the fault indicators require only a negligible resource overhead to keep the hardware costs as well as the energy consumption at a minimum while significantly improving the WSN’s reliability.

Security on the device and communication level was not in the focus of our work. However, security and dependability are integrated concepts [5], hence, increased reliability also typically influences security in a positive way.

### 1.3. Contribution, Methodology and Outline

The development of our sensor node is based on findings from the literature extended with results of our previous research (cf. [3,4,6,7]). Besides introducing the ASN(x), the contributions of this article include:

- a literature review on recent sensor node platforms,
- a taxonomy for faults in WSNs,
- a practical evaluation of the fault indicator concept proposed in [4], and
- the presentation of our embedded testbench (ETB), a Raspberry Pi hardware add-on that enables the analysis and profiling of embedded systems like sensor nodes.

Based on a tripartite experiment setup, we show the effectiveness of the ASN(x) in terms of node-level fault detection (especially soft faults) and its efficiency related to the energy consumption that is comparable with recent sensor nodes. The experiments consist of:

- an indoor deployment (i.e., normal operation in a controlled environment),
- an outdoor deployment (i.e., normal operation in an uncontrolled environment), and
- a lab setup running automated experiments with configurable environmental conditions such as the ambient temperature or the supply voltage, thus, forcing the sensor node in a form of impaired operation in a controlled environment.

The results confirm that our sensor node is capable of providing active node-level reliability based on the implemented fault indicators while keeping the energy consumption and the hardware costs at a minimum.

The remainder of this article is structured as follows. Section 2 elaborates on the sources and effects of faults occurring in sensor nodes and their respective detection techniques. A literature review on sensor node platforms with a focus on energy efficiency and/or node-level fault-detection capabilities published between 2015 and 2021 is presented in Section 3. Our sensor node platform, the ASN(x), and its components are discussed in Section 4. Section 5 describes our setup for the practical evaluation followed by results of the power analysis of the ASN(x) and the self-diagnostic measure evaluation in Section 6. Section 7 concludes this article and presents possible extensions and future research directions.

## 2. Faults in Wireless Sensor Networks

The deployment of large numbers of sensor nodes consisting of mainly low-cost components operated under uncontrollable environmental conditions poses a serious threat to the reliability of WSNs. Well-established reliability concepts such as hardware and/or software redundancy are mostly not applicable to WSNs due to the strictly limited resources of the sensor nodes [8]. As a consequence, faults in sensor networks tend to be the norm rather than an exception [9,10].

The detection of faults is often considered an outlier detection task and based on the sensor data only. This approach, however, suffers from a crucial problem: outliers do not need to be caused by faulty sensor nodes as they can also be the result of a rare but proper event in the sensed phenomena [4,11]. Additionally, faulty sensor nodes can report incorrect sensor values that may mimic non-faulty data [12].

Consequently, the effective and efficient detection of faulty sensor nodes is a challenging task. For this reason, this section discusses fault types appearing on sensor nodes and the severity they have on the network’s reliability followed by a presentation of related work on fault detection in WSNs.

### 2.1. Terminology

Although, the majority of works follow the terminology proposed by Avizienis et al. [5] which also serves as the basis for the notion of dependability defined by the *IFIP Working Group 10.4 on Dependable Computing and Fault Tolerance* (IFIP Working Group 10.4 on Dependable Computing and Fault Tolerance, refer to https://www.dependability.org/wg10.4/, accessed on 12 October 2021), the terms *faults*, *errors*, and *failures* are sometimes used inconsistently in the literature.

According to [5], a *fault* is a static defect in software or hardware components that can be either human-made (i.e., design fault), be related to the imperfections of the real world that affect the hardware (i.e., physical faults), or can be caused by the interaction with external components (i.e., interaction faults). In case of design faults, the term *bug* is commonly used. A fault is *active* if it leads to an error, that is, an incorrect internal state such as a deviation from correctness or accuracy; otherwise the fault is *dormant*. An error can propagate and ultimately lead to an observable deviation of the component’s behavior from its specification that is called a *failure*.

**Figure 2:**
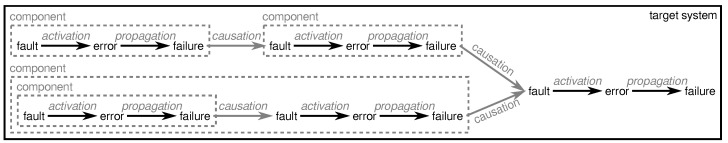
The fundamental chain of dependability & error propagation (after Figures 10 and 11 in [5]).

As depicted in Figure 2, a failure of one component can be the causation of a fault in a subsequent or superior component and can eventually lead to the failing of the target system (i.e., system failure). This effect is covered by the *fundamental chain of dependability* and is a crucial issue for reliability considerations. Nevertheless, the classification of whether an undesired effect counts as fault or failure depends on the actual focus of considerations, that is, where the system or component boundaries are drawn.

**Figure 3:**
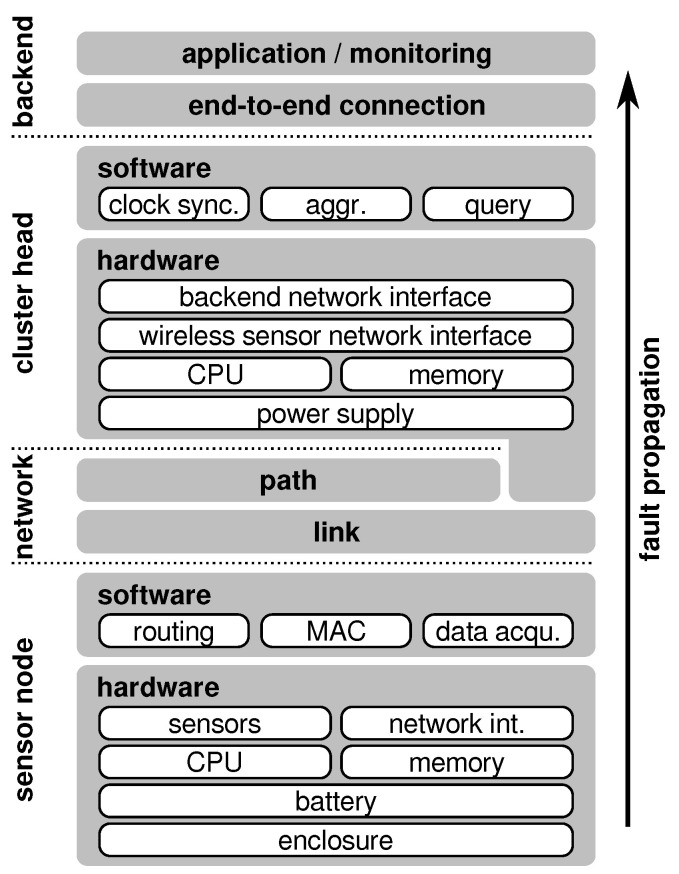
Fault propagation in a wireless sensor network (after Figure 1 in [18]).

The larger and more complicated a system is, the higher the probability of faults and, in turn, the higher the chance that a fault in an underlying component can lead to a system failure. In the case of a WSN, the situation is even worse as it usually consists of a large number of components (i.e., sensor nodes and cluster heads) that together form the system and contribute to the system’s functionality. As shown in Figure 3, faults in the sensor nodes can propagate through the network and, in the absence of counter-measures, can cause the system to operate incorrectly or even crash completely. For this reason, it is important to apply certain measures to prevent the propagation of component failures up to the system level and, thus, make the system fault-tolerant. Common practices include, for example, redundancy [13,14], fault detection and mitigation [15,16], or homeostatic approaches [17].

**Figure 4:**
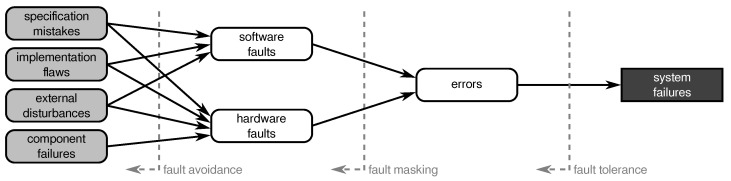
The cause-and-effect relationship of faults (after Figure 2 in [19]).

Measures to decrease the probability of having faults in a system are referred to as *fault avoidance*. Techniques to prevent active faults from causing erroneous systems states are denoted as *fault masking* and *fault tolerance* comprises actions to reduce the risk of errors propagating to failures (see also Figure 4).

Depending on the level where the fault-tolerant measures are applied, we can distinguish between:

- system-level fault tolerance,
- network-level fault tolerance, and
- node-level fault tolerance.

However, most often measures on all levels need to cooperatively work together to achieve a high degree of reliability. Nevertheless, depending on which level the measures are applied and where the focus of the system is laid (i.e., the boundaries) the terms *faults*, *errors*, and *failures* are sometimes confused and, thus, are used inconsistently in the literature.

In this article, we use the terminology of Avizienis et al. [5]. Our target system is the entire WSN as it is cooperatively responsible for the data acquisition. As a consequence, failures of the sensor nodes or their components are considered to be faults from a system-level perspective. To avoid confusion, within this article we use the term “WSN” to refer to the entire system while the term “network” refers to the interconnects between the sensor nodes and the cluster heads, respectively.

### 2.2. Wireless Sensor Network Fault Taxonomy

**Figure 5:**
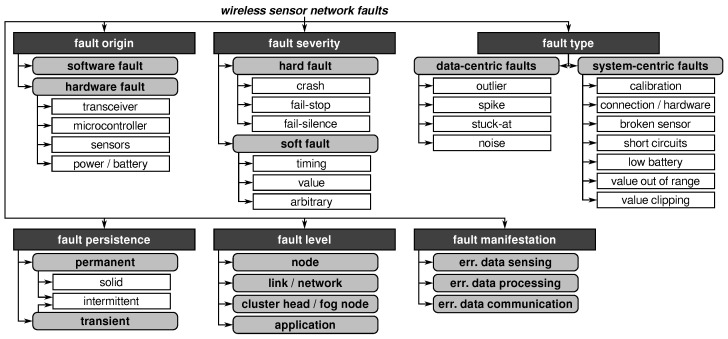
Wireless sensor network fault taxonomy.

The sources and manifestations of faults in WSNs are very diverse (cf. [5]). Faults can origin in different parts of the system and cause failure modes of distinct failure severities, that is, a faulty component does not always cause the system to fail in the same way. In the following, we discuss the diverse kinds of faults based on the taxonomy of wireless sensor network faults depicted in Figure 5.

While parts of this taxonomy are generally applicable, it is specially tailored to the characteristics of WSNs especially concerning their hardware components, network structures, and fault types commonly appearing in sensor networks. Such a classification scheme helps to develop appropriate countermeasures as it allows the identification of the relevant fault types, the components affected, and the level where the measures need to be applied.

Some of the categories (i.e., fault origin, severity, and persistence) are generally applicable to various kinds of systems. The categories fault type, level, and manifestations are system-specific and include unique attributes and characteristics of WSNs. However, some categories are not completely complementary as faults may combine features of different elements.

#### 2.2.1. Fault Origin

Wireless sensor nodes are embedded systems consisting of tightly integrated software and hardware components. While the software is usually considered as one single component, the hardware part can be divided into the radio transceiver, the MCU, the sensors, and the power supply (i.e., battery). Both, the software and hardware components can suffer from various faults where the manifestations depend on the actual origin of the fault. As shown in Figure 4, software mainly suffers from human-made faults such as specification or implementation mistakes (also called design flaws). Hardware components additionally have to cope with component failures due to physical faults.

Aside from supply voltage-related effects, especially the ambient temperature has shown to cause unpredictable behavior or defects in hardware components [9]. For example, high ambient temperatures accelerate the aging of the components that bring forward effects such as hot carrier injection (HCI), time dependent dielectric breakdown (TDDB), or negative bias temperature instability (NBTI). High temperatures further facilitate hardware-stress-related effects such as increased electromigration or the forming of metal whiskers.

While design flaws can be targeted with simulations or testing, physical faults caused by the imperfections of the real world cannot be adequately captured before the WSN’s deployment and, thus, runtime measures to enable fault-tolerance are needed.

#### 2.2.2. Fault Severity

Faults do not always cause the system to fail in the same way, neither concerning their manifestations nor the severity of their effects. While some faults may not even be noticeable, others can cause disruptions of the entire sensor network. In this context, two major groups of faults can be distinguished, namely *hard faults* and *soft faults*.

Hard faults include node crashes or the inability of a network participant to communicate with others such as fail-stop or fail-silence states. Such faults usually require human intervention to resolve the situation. For example, the authors of [20] found that bit flips in AVR-based sensor nodes mostly cause the node to crash. Sensor nodes deployed in harsh environments are especially susceptible to bit flips due to environmental disturbances. However, hard faulty network participants can normally be easily detected by their neighbors indicated by an absence of messages over a certain period.

Soft faults, on the other hand, are a notably greater danger to the data quality of a WSN. While hard faults usually result in missing data, soft-faulty components continue to report data, but with reduced or impaired quality. The effects of soft faults can range from deviations in the runtime behavior that can cause services to time out, over silent data corruption by incorrect data sensing or processing up to completely arbitrary effects. In addition, soft faults pose a great danger as such adverse effects are hard to detect by other network participants. As a consequence, corrupted or even arbitrary sensor readings can be propagated to the subsequent data processing resulting in wrong decisions or (counter-)actions. For this reason, especially soft faults are a serious risk to the reliability of WSNs and pose a crucial challenge for fault-tolerant networks.

#### 2.2.3. Fault Type

Faults appearing in sensor networks can also be described according to their manifestation in the sensor data and/or the system behavior. As a consequence, there are two views on the types of fault models for fault detection approaches as presented by Ni et al. in [10]. However, both views are not disjoint and most of the faults from one view can be mapped to faults of the other one (cf. Table IV in [10]).

The *data-centric* view describes faults by the characteristics they cause in the data behavior (diagnostic approach). This approach can also be used to describe faults where there is no clear explanation of its cause. Examples of data-centric faults are outliers, spikes or abrupt changes, stuck-at faults, or noise with a high variance.

The *system-centric* view, on the other hand, defines faults based on the effect certain flaws occurring in the system cause in the data it produces. One of the most common sources for system-related data distortion are depleting batteries of the sensor nodes or calibration faults of the sensors used [21]. But also hardware or connection failures (including short and open circuits) or environmental conditions such as a value out of sensor range (e.g., clipping) can cause faulty sensor data. However, in contrast to data-centric faults, the effects of system-centric faults depend on the actual system implementation such as the hardware components used.

#### 2.2.4. Fault Persistence

**Figure 6:**
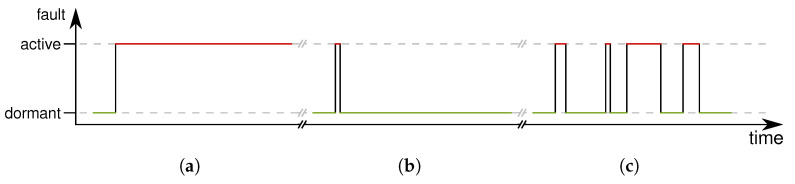
Fault categorization based on their persistence. (**a**) permanent/solid fault, (**b**) transient fault, (**c**) intermittent fault.

Another criterion to categorize faults is the persistence of faults. In this context, Avizienis et al. [5] defined two kinds of faults, namely *permanent faults* and *transient faults*. While the presence of permanent faults is assumed to be continuous in time (Figure 6a), the presence of transient faults is bounded in time (Figure 6b).

The persistence of faults can be further categorized based on their activation reproducibility. Faults with reproducible activation patterns are called “solid” (or hard) and those without systematically reproducible patterns are named “elusive” (or soft). Solid faults are the result of permanent faults. As discussed in [5], the manifestations of elusive (permanent) faults and transient faults are similar and, thus, are grouped together as *intermittent faults* (Figure 6c).

In sensor nodes, typical causes of permanent faults are physical damage or design flaws. Transient faults can additionally be the result of external circumstances such as interference. While solid faults have a permanent effect on the sensor nodes’ operation, the effects of intermittent faults happen sporadically and with varying duration, hence, often causing an unstable device operation.

#### 2.2.5. Fault Level

As depicted in Figure 3, faults happening on lower levels can propagate through the network affecting subsequent components in the data flow. Thus, faults can also be categorized based on the location where they happen (or the level, respectively). Fault tolerance measures can thereby focus on particular parts of the network or several components of the data (sub)flow.

However, based on the level where the measures are applied, the basic approaches may differ. While certain node faults are most efficiently detected by self-check techniques, faults in the links between network participants are better to be captured with group detection or centralized approaches. Additionally, the higher the level of the network the more resources are usually available and, thus, the more sophisticated measures can be applied. For example, monitoring and fault diagnosis techniques applied on the cloud level can normally utilize a certain level of processing power, have significantly more memory available, and do not need to consider an energy-efficient operation. Devices in the edge layer, on the other hand, can only use lightweight techniques that do not contravene the resource limitation of those. As a result, the fault level considered is an important criterion for selecting appropriate countermeasures.

#### 2.2.6. Fault Manifestation

Faults can also be described based on the functionality they impair. The basic functionality of a sensor network comprises:

- the measurement of certain physical quantities (i.e., data sensing),
- the (pre)processing of the acquired data (i.e., data processing), and
- the forwarding of these data via the network (i.e., data communication).

A fault can affect one or more of these functionalities, possibly with different severity.

An erroneous data sensing can be caused, for example, by sensor hardware failures, electrical connection issues, or in case the sensed phenomena is outside of the sensor’s measurement range (or in its saturated area; refer to Figure 2 in [10]). Erroneous data processing can be caused by design faults like software bugs or by physical faults affecting the processing units such as the sensor nodes’ MCU. Errors in data communication can stem from a wide range of sources. Aside from flaws in the communication protocols, also network attacks or environmental factors can hinder the proper communication between participants. Especially environmental factors such as the ambient temperature have been shown to influence (or even temporarily prevent) the communication within the network (cf. [22,23]).

### 2.3. Faults vs. Anomalies

The detection or diagnosis of faults generally suffers from one crucial problem as quoted from Ni et al. in [10]:

“*Unless ground truth is known or given by something with high confidence, the term fault can only refer to a deviation from the expected model of the phenomenon.*”

Thus, in the absence of ground truth, fault detection mainly refers to the detection of deviations from expected behavior, that is anomaly detection [21]. For this reason, often anomaly detection techniques are applied in fault detection approaches. A recent meta-survey on anomaly detection in WSNs and taxonomy for respective techniques can be found in [3]. Such techniques are useful in detecting data-centric faults by identifying anomalous patterns in the sensor data.

**Figure 7:**
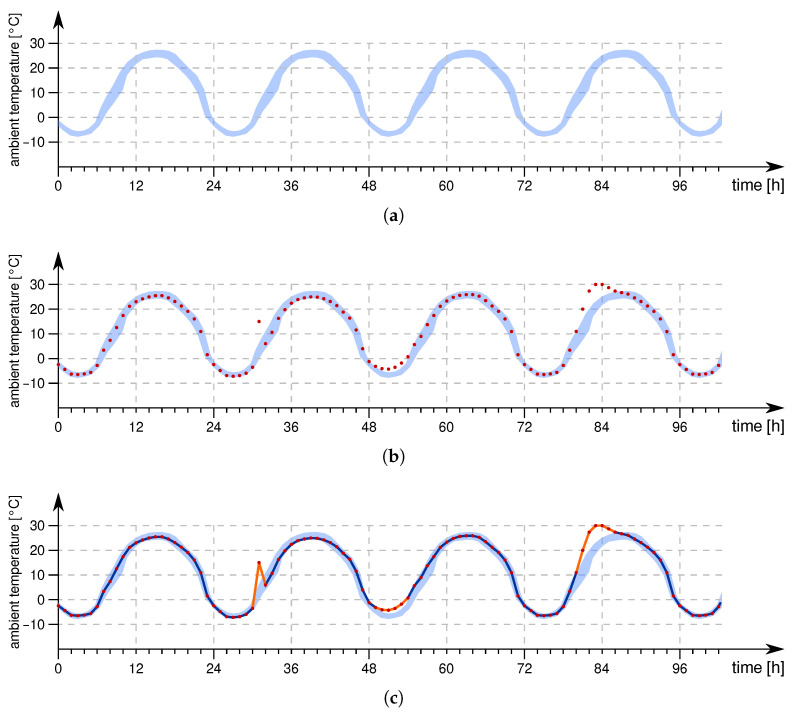
Anomaly detection in an environmental monitoring example. (**a**) Derived model of the “normal” behavior, (**b**) Continuous sensor value updates, (**c**) Data anomalies: soft faults or proper events?

As an example, consider a WSN used to continuously monitor and report the environmental conditions of a certain area of interest, for example, the temperature. Based on prior domain knowledge, available historic data, or after collecting sufficient data a model of the expected (or normal) behavior of the temperature curve can be derived (see the blue-shaded area in Figure 7a). In outdoor applications, the temperature normally follows a diurnal pattern with day and night cycles. This process is usually done on a central point with sufficient resources such as a cloud server. As the WSN continues to monitor the temperature, continuously new data instances become available depicted as red dots in Figure 7b. When analyzing the newly arriving data regarding the expected behavior (i.e., the “normal” model) certain deviations can be found in the reported data. Regarding a data-centric view, these deviations can be manifested as drifts, offsets, or outliers as shown by the orange regions in Figure 7c.

The big question now is whether these anomalies in the sensor data stem from proper but rare events in the monitored phenomena or are deviations caused by faults in the sensor network (i.e., soft faults). On the higher level of the data processing chain (e.g., the cloud) both effects are hard to distinguish, or even impossible if no further information is available. For example, a spike in the temperature curve may be a strong indicator of a fault, but can also be caused by direct sunlight that hits the area where the temperature is measured. So far, the distinction between outliers caused by proper events from those resulting from faults has only been sparsely addressed [24] and, thus, is within the focus of this research.

### 2.4. Fault Detection in WSNs

Faults are a serious threat to the sensor network’s reliability as they can significantly impair the quality of the data provided as well as the network’s performance in terms of battery lifetimes. While design faults can be addressed during the development phase, it is close to impossible to derive proper models for the effects of physical faults. Such effects are caused by the interaction of the hardware components with the physical environment and occur only in real systems. For this reason, they can not be properly captured with well-established pre-deployment activities such as testing and simulations. Hence, it is necessary to incorporate runtime measures to deal with the multilateral manifestation of faults in a WSN.

Fault tolerance is not a new topic and has been addressed in numerous areas for a long time already. Like WSNs, also systems used in automotive electronics or avionics mainly consist of interconnected embedded systems. Especially in such safety-critical applications where system failures can have catastrophic consequences, fault management schemes to mitigate the risks of faults are a must-have. Consequently, the automotive functional safety standard ISO 26262 provides methods and techniques to deal with the risks of systematic and random hardware failures. The most commonly applied concepts are hardware and software redundancy by duplication and/or replication [25]. Similarly, also cyber-physical systems (CPSs) used in, for example, industrial automation commonly use duplication/replication to enable a certain level of resilience [13,14].

However, redundancy-based concepts often interfere with the requirements of WSNs as they require a significant overhead regarding the system sizes and costs, but especially concerning the energy consumption [8]. Therefore, such concepts are hardly suitable for WSNs. Fault management schemes suitable for sensor networks have to be energy efficient, provide a suitably high fault detection accuracy, need to be able to cope with the characteristics of the wireless network (e.g., message delay), and should not suffer from scalability issues [15].

In the following, an overview of the basic detection strategies of fault management schemes for WSNs published in the recent past is presented. The majority of approaches can be classified into three main categories based on their general detection strategy:

1. sensor data analysis (see Section 2.4.1),
2. group detection (see Section 2.4.2), and
3. local self-diagnosis (see Section 2.4.3).

For a detailed survey on fault detection and tolerance schemes applied to WSNs, we refer an interested reader to the literature reviews presented in [15,26].

#### 2.4.1. Sensor Data Analysis

One way to detect faults in a sensor network is to analyze the data reported by the sensor nodes. Faults often manifest as anomalies in the sensor data, hence, anomaly detection approaches are commonly used [3]. Since faults can have different causes and result in effects of variable duration and impact, many of the data-oriented detection approaches leverage correlations available in the sensor data (e.g., temporal, spatial, or functional) as a substitute for missing ground truth. However, to consider temporal correlations also previous sensor data are required (i.e., the history). Spatial correlations, on the other hand, rely on the data from various sensor nodes within a certain neighborhood. As a result, many of the sensor data analysis approaches are run centrally on systems with higher resources such as the cluster head or even in the cloud layer.

Most of the data-oriented approaches can be categorized into:

(i) statistics-based,
(ii) rule-based,
(iii) time series analysis-based, or
(iv) learning-based methods.

To cover a broader spectrum of faults, to improve the detection rate, or to lower the false alarm rate, hybrids can be used that combine different methods. An overview of data-based fault detection approaches can be found in the outlier detection survey presented in [27] or the review on noise or error detection approaches given in [28].

(i) In statistics-based detection methods commonly metrics such as the mean, the variance, or the gradient of the sensor data are considered for outlier detection [29], but there are also more sophisticated approaches that, for example, apply the Mann-Whitney U statistical test or the Kolmogorov-Smirnov test [30] to identify permanent, intermittent, and transient irregularities in the sensor data [31] as well as 3σ-based techniques using historical data and the measurements of neighboring nodes [32].

(ii) Rule-based methods derive heuristic rules and constraints for the sensor readings often by exploiting domain or expert knowledge. Such approaches can range from adaptive thresholds of the sensor data [33] over signature-based fault detection [34] up to applying distributed state filters on the sensor data [35].

(iii) Time series analysis-based methods leverage temporal correlations in timely ordered data of one or more sensor nodes collected over an internal of time to predict the expected values for future data (cf. [36]). An anomaly is then assumed to be the deviation of the measurements and the predicted values [37].

(iv) Another possibility to infer a model of the “normal” sensor data is the use of learning-based methods. Based on the derived model, deviations of the actual sensor readings from the expected values can then be detected. Thereby, particularly neural networks [38,39] and support-vector machine (SVM)-based detection approaches [40] have shown to be suitable in identifying anomalous sensor readings, especially when being augmented with statistical features as described in [41]. But also approaches based on decision trees have been proposed for fault detection [42].

However, most data-centric detection approaches consider the sensor nodes as black boxes and neglect information available on a node level. As a consequence, such approaches often suffer from difficulties distinguishing anomalies caused by faults from actual events in the monitored phenomena. In addition, several approaches are not generally applicable, because they require expert/domain knowledge that is often not available or base their detection technique on application-specific assumptions.

#### 2.4.2. Group Detection

The detection of faults based on the spatial correlation of sensor data forms the basic principle of the second category of fault detection schemes, the group detection-based approaches. Such approaches can either be run centrally on, for example, the cluster head or distributed on several (or even all) network participants. In some approaches, additional monitoring nodes with higher resources are added to the network to observe the behavior of their local neighbors. However, group detection approaches commonly rely on three major assumptions:

(i) the sensor nodes are deployed densely (i.e., the difference in the measurements of two error-free sensor nodes is negligibly small),
(ii) faults occur rarely and without systemic dependencies (i.e., the number of faulty nodes is much smaller than the number of non-faulty nodes), and
(iii) faults significantly alter the sensor data (i.e., a faulty sensor reading significantly deviates from proper readings of its local neighbors).

Additionally, some approaches assume that faults occurring in the network are permanent (cf. [43]), hence, transient and intermittent faults are not considered. Aside from the approaches’ architecture (i.e., centralized vs. distributed), the approaches differ in the way they decide on faulty readings (e.g., voting [44], aggregation [45]) and in the information used for their decision (e.g., sensor readings, battery levels, link status). For example, the battery level in combination with the link status can be used to define the sensor nodes’ state of health that is then shared with the node’s neighbors [46].

To detect faults, the approaches apply (spatial) anomaly detection methods [47], consider mutual statistical information of the neighbors [11], or use a (dynamic) Bayesian classifier [2]. The approach proposed in [48] extends a dynamic Bayesian network with a sequential dependency model (SDM) separated in time slices where spatial correlations can be exploited in a single time slice and temporal dependencies can be treated by exploiting time slices of different nodes. Another example of group fault detection is the algorithm presented in [49] that incorporates physical constraints of the monitored phenomena based on which the Kalman filter estimation value of adjacent nodes is calculated. As stated in [3], especially artificial immune system (AIS)-based approaches have properties beneficial to anomaly and fault detection in WSNs such as the distributed AIS-based fault diagnosis algorithm proposed in [50].

Although showing good detection rates, group-based approaches suffer from major drawbacks stemming from the above-mentioned assumption. For instance, to ensure that the distance between two neighboring nodes is always small enough to have negligibly small differences in their measurements would require a large number of nodes and, thus, would be expensive and the network may suffer from scalability issues. Also, the assumptions on the faults cause difficulties as faults have shown to appear frequently in WSNs and their effects may be subtle such as silent data corruption. But most of all, group detection approaches often require a high communication overhead due to the message exchanges between the neighboring nodes. As a consequence, the energy consumption of the nodes is significantly increased resulting in shorter battery life.

#### 2.4.3. Local Self-Diagnosis

The third main class of fault detection approaches is executed on the nodes locally. In contrast to the above-presented sensor data analysis and group detection concepts, the local self-diagnosis is applied close to the source of faults where node-level information can be used for better fault detection. Since these approaches exploit the nodes’ internal information (i.e., node-level data) they can be seen as a form of glass-box (or white-box) runtime testing. In addition, such approaches do not suffer from scalability problems as the detection is run on the nodes locally.

One possibility for fault self-diagnosis is to run lightweight data-centric techniques on the nodes that detect statistical deviations in the node’s measurements (i.e., mean and variance) or perform low-level anomaly detection similar to the methods described in Section 2.4.1. However, some researchers suggest including node-level information aside from the sensor readings to analyze the node status at runtime [3,51]. Several works have been presented in the last years that incorporate such node-level information in their approach, but so far most of them use rather simple checks based on the remaining battery charge (measured by the battery voltage level) or the nodes’ link status (e.g., received signal strength indicator (RSSI) or signal-to-noise ratio (SNR); cf. [18,52,53]). In case the nodes are running an operating system (OS) also metrics such as the central processing unit (CPU) load (i.e., number of cycles executed by the MCU), the memory consumption, or the execution time available from the OS have been included in the detection [54,55]. Aside from information already available in software, it is also possible to extend the sensor nodes with specific hardware for fault diagnosis, for example, using a secondary MCU that supervises the main MCU [56] or a current monitor that allows detecting faults specific to certain sensors [12].

As with data-centric and group detection approaches, also self-diagnostic techniques are challenged by the limited resources of the sensor nodes. But in the case of local self-diagnosis, the situation is even worse as the approach is applied on all nodes and, thus, has to be lightweight and energy-efficient. If additional hardware is required also the cost factor has to be kept in mind. As a result, the majority of approaches so far rely on simplistic checks of, for example, the residual energy or OS-related metrics. Although being mentioned before, the incorporation of additional node-level information to augment the fault detection on a node level has not been analyzed, yet. For this reason, we present a novel detection approach that extends the idea of local self-diagnosis as described in Section 4.5.

## 3. Sensor Node Platforms

As discussed before, faults are a serious threat to the proper functioning of a WSN. Faults can thereby originate in different places in the WSN and can, in case no appropriate counter-measures are applied, propagate through the network and can eventually lead to system failures. Therefore, it is important to apply suitable measures to mitigate the effects on all levels and layers of the WSN.

On the lowest layer, WSNs consist of interlinked low-power embedded systems responsible for sensing certain physical quantities that are commonly referred to as *sensor nodes* or sometimes also called *motes* (in this context, the term “mote” refers to sensor nodes of particularly small size), see Figure 1. The sensor nodes are key components of the WSN and have a significant impact on the network’s performance and accuracy. Selecting proper hardware components for the sensor nodes is crucial to ensure a reliable operation (even under harsh environmental conditions) while providing data of high quality. This, however, is not trivial as the sensor node design is challenged by the tradeoff between low-power operation and sufficient computational performance as well as using low-cost components whilst being small in size [57]. Yet, the most limiting factor for sensor node design is the strictly limited energy budget as sensor nodes are usually battery powered and energy harvesting is not always possible or feasible.

Basically, there are three options for the sensor node development, namely:

(i) to build sensor nodes from scratch (*custom nodes*),
(ii) to utilize a generic embedded platform (*semi-custom nodes*), or
(iii) to use an available sensor node platform (*commercial* or *academic nodes*).

(i) *Custom sensor nodes* are designed for a particular application and, thus, offer the highest degree of specialization to the corresponding requirements. Their development requires a considerable amount of time and resources as well as a certain degree of expertise. Aside from selecting appropriate hardware components, considerations on the power supply, and developing the printed circuit board (PCB), also the software consisting of an OS or middleware, the sensor drivers, and the communication drivers as well as the respective toolchain need to be prepared.

(ii) Alternatively, the sensor nodes can be developed in a *semi-custom* fashion with a generic embedded platform (i.e., development and breakout boards) extended with application-specific hardware such as a radio transceiver and sensors. Such approaches usually require less development time than custom sensor nodes and often result in cheaper hardware costs as many embedded platforms are available at low prices. In addition, many of these embedded platforms are supported by a large community providing software drivers and example codes. The most-known generic embedded platforms include *Arduino*, *BeagleBoard*, *Raspberry Pi*, *Teensy*, *Espressif* (*ESP*), and *mbed*. However, these platforms usually target a wide range of applications, hence, they are not specifically designed for low-power sensor node operation. As a result, such platforms often offer high computational power and certain onboard circuitry supporting the development process (e.g., light-emitting diode (LEDs)) at the cost of a higher overall power consumption (cf. [6]).

(iii) In the last two decades, a great number of wireless sensor nodes have been developed by universities, research institutions, or companies. Some of these nodes are (or have been) commercially available or were even made public as an open-source project. In the research community, probably the most-famous sensor nodes are the *Berkeley motes* including the well-known *Mica* or *Telos* motes [58,59]. The advantage of using such ready-to-use nodes are the significantly shortened development times and the availability of the core software components in combination with hardware tailored for the (ultra) low-power requirements of wireless sensor nodes. On the downside, these nodes are often specific to certain use-cases offering moderate flexibility and several sensor nodes are not publicly available or not available anymore.

### 3.1. Basic Components

Sensor nodes differ in the hardware components used, their size and weight, the supported power sources and battery lifetimes as well as the sensors available or the analog/digital interfaces, respectively. The choice of suitable hardware components is crucial as they essentially determine the nodes’ functionality and operational characteristics including their energy efficiency and reliability. Regarding the latter, the components influence the probability and nature of faults that can impair the nodes’ proper function.

**Figure 8:**
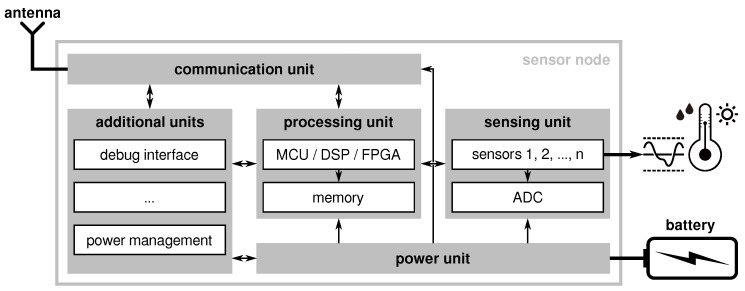
Basic components of a wireless sensor node (after Figure 1 in [60]).

The functionality of a typical sensor node includes (i) the measurement of certain physical quantities, (ii) the (pre-)processing of data, (iii) the forwarding of their data over wireless links, and (iv) the energy-efficient operation to ensure long battery lifetimes. Consequently, the hardware of most sensor nodes can be divided into four basic blocks as depicted in Figure 8, namely:

(i) a set of sensors,
(ii) a processing unit (optionally with external memory),
(iii) a radio transceiver, and
(iv) a power unit with a power source (i.e., a battery).

Additionally, the sensor nodes can be equipped with additional units such as a debugging interface to support the software development or with external power management capabilities on top of the power units.

(i) The *sensor unit* is responsible for acquiring sensory information of certain physical quantities. Depending on the application requirements, different types of sensors for various physical quantities are available. These sensors not only differ in the quality of the provided measurements (i.e., resolution, accuracy, conversion time) but also in the way to provide the measurement to the subsequent processing unit. Thereby, two basic kinds of sensors can be distinguished, namely sensors available as integrated solutions that provide their measurements via a digital interface (e.g., universal synchronous/asynchronous receiver-transmitter (USART), serial peripheral interface (SPI), or inter-integrated circuit (I2C)) and sensors (or sensory circuits) that output an analog signal proportional to the measured physical quantity. The latter requires an analog-to-digital converter (ADC) to make the measurements available to the processing unit that can be either an on-chip peripheral of the processing unit or a separate hardware component.

(ii) The *processing unit* is the heart of the sensor node and takes care of gathering the measurements from the attached sensors, prepares these values for transmission (possibly including some pre-processing like normalization, conversion, or plausibility checks), and eventually forwards the data via the communication unit. While most sensor nodes use an microcontroller unit (MCU) as a processing unit sometimes extended with external flash memory, there are also solutions based on digital signal processors (DSPs), field-programmable gate arrays (FPGAs) or even highly-integrated systems-on-a-chip (SoCs) with multicore architectures [57]. Aside from a shorter time-to-market, MCU-based nodes are beneficial due to their low prices and comparably low power consumption. The majority of MCU-based sensor nodes currently either use an 8-bit AVR ATmega, a 16-bit TI MSP430 or a 32-bit ARM Cortex-M0/M3 MCU [61]. Nevertheless, in the last years a shift towards SoC-based nodes has been noticed, where the processing and communication unit are both integrated into a single chip [58].

(iii) The choice of the communication unit depends on the transmission medium and the communication system to be used. Even though the majority of WSNs use radio frequency (RF)-based communication (e.g., using radio transceivers in the license-free industrial, scientific and medical (ISM) bands at 868/915 MHz and 2.4 GHz), some applications require other forms of communication such as ultrasonic-based systems used in submarine WSNs (cf. [62]). Except for multimedia WSNs, the majority of sensor networks use moderately low data rates of up to 250 kbit/s. Especially monitoring applications such as environmental monitoring usually require the transfer of comparably small network packages that are transmitted at lower data rates to keep the power consumption at a minimum. Depending on the range of the communication link, the radio transceivers can have an external, PCB, or chip antenna attached. A summary of some commonly used RF modules can be found in [63] and an overview of wireless standards and technologies often used in WSNs is given in [61].

(iv) The power unit is responsible for providing the sensor node’s components with power that is usually supplied by a battery. If the node uses energy harvesting (e.g., solar cells), the power unit additionally needs to control the charging cycles of the battery. Especially for sensor nodes without energy harvesting, the choice of the power unit is often straightforward. Several nodes have the battery directly connected to the supply rail of the node’s components. To avoid unintended effects of a depleting battery (i.e., sinking battery voltage) such nodes usually rely on the brown-out detection of components that disables them in case of a too low supply voltage. Other sensor nodes cope with the effects of a possible undervolting on a higher level (cf. [64]). However, the majority of sensor nodes use linear regulators to ensure a stable supply voltage, but at the cost of bad energy efficiency. Especially ultra low power (ULP) sensor nodes tend to exploit the high efficiency of DC/DC converters where modern solutions only require a few additional (passive) components. The particular supply options and their pros/cons are discussed in more detail in Section 4.3.

### 3.2. Related Sensor Node Platforms

To ensure a high quality of the data provided and a reliable operation of WSNs, the sensor nodes need to be energy-efficient and fault-tolerant at the same time. There is a trade-off between these two characteristics as measures to increase the reliability usually imply an energy-overhead that is proportional to the complexity of the taken measure. Most often the reliability is implemented on a network level and the focus of the sensor node design is on energy efficiency rather than fault tolerance.

In the following, a literature review on recent sensor node platforms is given that extends the surveys presented in [57,59,61,63]. It focuses on sensor nodes that either support high energy efficiency (i.e., ULP operation), include techniques for node-level fault tolerance (i.e., self-diagnostic measures), or both. The review was conducted by searching the publication databases *IEEE Xplore*, *ACM Digital Library*, *ScienceDirect*, *Springer Link*, and *Google Scholar* for conference and journal papers published in the years 2015–2021 using the search string (items are AND connected; database searches included all metadata available):

- “WSN” OR “wireless sensor network” OR “sensor network” OR “sensor”
- “node” OR “mote” OR “board” OR “platform”
- “design” OR “development” OR “implementation” OR “concept”
- “reliability” OR “resilience” OR “fault tolerance” OR “fault diagnosis”

An overview of the sensor nodes found, their year of publication, and their characteristics are given in Table 1. In addition, several WSN deployments used sensor nodes based on Arduino boards such as the *Nano* [65,66] or *Uno* [67,68,69,70,71]. The Arduino boards, however, are not suitable for low-power sensor nodes as the additional onboard circuitry such as user-definable LEDs and universal serial bus (USB)-to-USART bridges consume too much energy [6,72]. For this reason, they are not further considered in this article.

The table lists the core components of the sensor nodes, that are, the MCU including its specifications in terms of CPU architecture, clock frequency (F_CPU_), and available memory as well as the radio transceiver and used communication standards. Also, information on the nodes’ supply voltage for the core components (V_core_), the supported input voltage range (V_bat_), and the typical power consumption in the active and power-saving modes are listed (as most authors present the current consumption of their sensor nodes rather than the power consumption, we calculated the corresponding power by multiplying the given current values with the nodes’ core voltages to allow for better comparison). However, the transmit and receive power consumption of the radios are neglected in the table as the values depend on the actual radio settings (e.g., transmit power). Information on these values can be found in the corresponding datasheets of the radio transceivers. Additionally, the table lists the *voltage regulation* technique where:

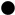
refers to nodes using a DC/DC converter,
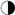
denotes nodes using a linear regulator (e.g., low-dropout regulator (LDO)), and
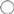
highlights nodes that have the battery directly connected to the core supply rail.

Next, the columns *energy-efficiency* and *self-diagnostic* state to which extent energy-efficiency was considered in the node design and whether self-diagnostic measures are included, respectively. In the *open source* column, the extent to which the sources of the nodes’ design and software are publicly available are highlighted where:

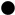
means that all related information is publicly available,
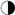
refers to nodes where only parts are available (mostly the software), and
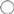
shows that no information has been made publicly available.

Last, the availability status of the nodes (i.e., whether the nodes are (still) available on the market) and the price of one sensor node are listed. For commercial nodes, the price refers to the cost of one node on the market while for nodes presented in academic papers the cost estimation of the authors is stated. However, in both cases, the actual costs can vary depending on the distributor of the nodes or hardware components as well as the PCB manufacturer in the latter case. Also, some nodes come equipped with several sensors while others provide the baseboard only. Therefore, the provided values shall be considered as a reference value for coarse comparison.

In our review, we found that especially the energy characteristics stated by some authors have to be taken with care as in some cases only the consumption of single components (sometimes just taken from the corresponding datasheets) are stated rather than the actual consumption of the board including peripherals and passive components. Also, the information provided in some of the surveys is incorrect or at least questionable, especially if the source of information is missing.

The focus of this article lies on energy-efficient and/or node-level fault-tolerant sensor nodes. Therefore, sensor nodes focusing on energy efficiency and their power-saving approaches are discussed in Section 3.2.1 and nodes enabling self-diagnostics to enhance the WSN’s reliability are presented in Section 3.2.2.

#### 3.2.1. Energy-Efficient Sensor Nodes

The overview of sensor nodes in Table 1 reflects the importance of energy-efficiency in WSNs. Except for two designs, energy efficiency was at least partly considered in all nodes. Thereby, two main design criteria are important to ensure energy-efficient operation, namely:

(i) the duration of the active and the sleep phases (i.e., duty-cycling) and
(ii) the power consumption in both phases (i.e., energy-efficient hardware).

(i) Usually, the hardware components such as the MCU, the radio transceiver, and (where possible) also the sensors are kept in an active state for as short as possible. The rest of the time the components are put to a power-saving or sleep mode to save energy ([95]). In both states, the power consumption depends on the hardware used in combination with board assembly-related factors (i.e., passive components) and, in case used, OS-related characteristics. Consequently, the power consumption needs to be measured on a real prototype as the sum of the datasheets’ values is usually much lower than the reality.

Depending on the amount and type of sensors, the complexity of the data processing, and the communication standard, the active time is markedly smaller than the duration of the power-saving phase and is usually in the range of several milliseconds up to a few seconds. Hereby, also the hardware components have an impact on the duty-cycling as, for example, some sensors require a certain conversion time that can significantly prolong the active phase (e.g., the temperature measurement of the DS18B20 sensor takes up to 750 ms). The sleep time, on the other hand, depends on the application requirements and is commonly in the range of several seconds or minutes (up to a few hours in rare cases). Thus, the energy spent in power-saving mode commonly dominates the overall power consumption [58]. In this context, previous studies [96] found that one of the main contributors to active power consumption is wake-up energy. During the wake-up, the hardware needs to be re-started and possibly re-configured for proper functioning. Additionally, the device may need to re-connect to the network. As a result, the authors of [96] concluded that the length of the sleep phase in IEEE 802.11-based WSNs should be greater than 30 s with larger values resulting in better energy efficiency. Similar effects appear in other connection-oriented protocols such as Zigbee as shown in the results of our practical evaluation in Section 6.1.

(ii) Aside from a suitable active/sleep schedule, also the choice of hardware affects the sensor node’s energy efficiency. Most sensor nodes utilize low-power components (e.g., MCU or SoC) that have a comparably low power consumption in the active state often in combination with the support of energy-saving (e.g., disabling of unneeded on-chip peripherals) and power-down possibilities (i.e., sleep modes). Regarding the latter, the power consumption of the digital circuits (mostly CMOS-based) mainly consists of a static fraction caused by a certain leakage current, a dynamic part resulting from changes in the charge of the (usually parasitic) capacitances of the circuitry, and a transient short circuit power dissipation during the switching. However, the overall power dissipation is dominated by the dynamic part that has a linear dependency on the clock frequency and a quadratic dependency on the supply voltage. To lower the power demands of the circuitry both the frequency and the supply voltage may be decreased (down to certain thresholds). The runtime adjustment of the frequency is commonly done via dynamic frequency scaling (DFS) schemes and the adaptation of the supply voltage by dynamic voltage scaling (DVS) techniques. While DFS is often provided by, for example, the MCU/SoC, DVS usually requires additional on-board circuitry. In the past, DVS approaches applicable to sensor networks have been proposed that adjust the supply voltage level based on the system needs down to the minimum threshold specified by the manufacturer [97]. There is also so-called “active undervolting” where the supply voltage is even lowered below the specified minimum voltage level to further decrease the power consumption ([64]).

Besides, a suitable hardware selection of the sensor nodes is also relevant for leveraging the sleep modes of the processing unit (i.e., MCU). Most recent MCUs provide different power-down modes with different levels of saving potentials by deactivating the clock source for specific on-chip components or even the entire MCU. This, however, raises the need for external components able to wake the MCU up from its deep sleep. Most often low-power real-time clocks (RTCs) are added to the nodes’ design that can wake the MCU, for example, via an external interrupt. In such a case, the RTC can easily become a single point of failure as a missing wake-up signal can cause the sensor node to never wake up again. Therefore, the chosen wake-up source and its consequences on the node’s reliability need to be carefully considered.

Coming back to the overview on energy-efficient sensor nodes, we found that the majority focuses on using (ultra) low-power components in their design [72,89,90,91,93]. Some authors additionally took the passive hardware components required for proper functioning into their considerations [72,91]. Surprisingly, the majority of nodes found use linear voltage regulators [76,78,79,82,83,84,87,88,94] or even no regulation at all [72,73,75,80,86,91,92,93] instead of low-power DC/DC converters [54,74,77,81,85]. Especially if the battery is directly connected to the hardware components’ supply rail unintended effects (including soft faults) can easily happen at the end of the battery life due to a (passive) undervolting of the components ([7,64]). Also, the operating frequency of the MCU has only been discussed in a minor number of designs [72,83,84,92,93] by keeping the frequency as low as possible. Enhanced energy-saving approaches such as DVS (or active undervolting) were only considered in [64] (a possible explanation follows in Section 3.2.2). The impact of the software design on the nodes’ overall energy efficiency was only presented in a few works [72,91] as the majority focused on the hardware design and left the software to the application developer.

#### 3.2.2. Self-Diagnostic Sensor Nodes

While energy efficiency was considered in almost all sensor node designs found in the literature review, node-level reliability by implementing self-diagnostic measures was only partially treated in two cases [54,84]. All other designs relied on the implementation of reliability measures (e.g., fault detection and/or tolerance) on a network level rather than the node level. That is, the effects of faults occurring in sensor nodes have to be mitigated by other network participants before the affected data reach the subsequent data analysis. But dealing with node faults on the network or even cloud level faces serious issues concerning the detection of soft faults such as silent data corruption (see Section 2.4) and, thus, incorporating node-level information in the fault detection approaches is inevitable ([3,4]).

In the sensor nodes proposed by Raposo et al. [54], a sensor node monitoring agent is deployed in the firmware of each sensor node that collects certain runtime metrics provided by the OS or the firmware such as the CPU load (i.e., number of cycles executed by the MCU), the execution time, and the energy consumed (based on the approach proposed in [16]). Aside from these data, no node-level self-diagnostics were applied.

In the approach proposed by Kulau et al. [64] the primary focus is on energy saving by active undervolting. Thereby, the sensor nodes’ supply voltage is decreased below the minimum voltage threshold suggested by the manufacturer down to the lowest value that allows a proper operation. This value, however, depends on several factors including manufacturing-related characteristics as well as the ambient temperature. As a result, the voltage level can drop below the lowest operational point and the node stops working. To remedy such cases, the used INGA nodes [84] are equipped with a secondary MCU supplied by a constant voltage in the safe region. This secondary MCU monitors the primary MCU using spot tests and resets the supply voltage in case an error is detected, hence, forming a type of node-level fault detection. The spot tests applied are matrix multiplications checking the proper functioning of the primary MCU’s arithmetic logic unit (ALU). However, this approach has two main drawbacks that can endanger the WSN’s reliability, namely:

1. the secondary MCU can be impaired by faults, too, and
2. checking the primary MCU’s ALU only is insufficient to ensure reliability.

Regarding the latter, our previous research [7] showed that such a spot-test needs to take all on-chip peripherals into account since, for example, the minimum supply voltages for the ADC or the USART interface are notably higher than those of the ALU. There are voltage levels where the ALU properly works but the ADC and USART do not and, thus, the system is in a state susceptible to soft faults.

## 4. ASN(x)—An AVR-Based Sensor Node with Xbee Radio

Applying fault detection on a node level is crucial to improve the WSN’s overall reliability. Nevertheless, the majority of related sensor nodes found in our literature review enable low-power operation but do not offer any node-level fault detection capabilities. This, however, has major drawbacks as presented in Section 2.4. To improve the detection of node-level faults and allow one to distinguish between faults and proper events, we argue that it is inevitable to incorporate self-diagnostic measures into the sensor nodes’ design.

The development of a sensor node from scratch is a complicated task that takes much time and entails many pitfalls. For this reason, researchers often utilize ready-to-use sensor node platforms ([6]). As presented in Section 3.2, various commercial products from different manufacturers have been presented over the years. While these node platforms are normally highly optimized and are proven in practical use, they are often limited in their usage (i.e., tailored for specific applications), require specific toolchains or programming languages, are difficult to acquire (limited availability on the market), or are simply too expensive (refer to Table 1). Additionally, several custom sensor nodes have been used by researchers in the literature. However, most of them are not publicly available, use obsolete components, or lack support (especially after the respective research projects have ended).

Therefore, finding a suitable platform for WSN deployments is not easy but is necessary for practical research. An ideal platform should be open-source to offer transparency, be available to everyone, and should enable to be modified based on application-specific needs. Additionally, such a platform should not only be energy-efficient but should also incorporate measures to ensure a reliable operation by enabling node-level fault diagnostics.

Consequently, we developed the *AVR-based Sensor Node with Xbee radio*, or short ASN(x). The ASN(x) is a wireless sensor node platform, that:

- enables active node-level reliability by incorporating self-check capabilities,
- offers an energy-efficient operation especially suitable for monitoring applications,
- is versatile regarding its usage (i.e., modular expandability),
- is based on current components that are highly available on the market,
- is comparably cheap (about $50 per node including the radio), and
- is completely open-source published on Github under the MIT license.

**Figure 9:**
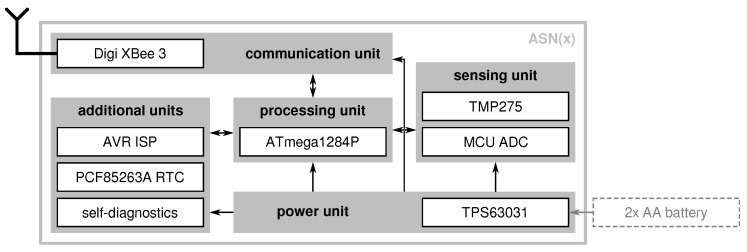
Basic components of the ASN(x).

As depicted in Figure 9, the ASN(x) (the information in this article refer to the hardware revision v1.3 of the ASN(x)). incorporates the basic components of a wireless sensor node presented in Section 3.1 (see also Figure 8), that are:

- a processing unit (see Section 4.1),
- a sensing unit (see Section 4.2),
- a power unit (see Section 4.3), and
- a transceiver unit (see Section 4.4).

Aside from an onboard temperature sensor (TMP275), the ASN(x) can be freely extended with application-specific sensors via extension headers with several general purpose input/outputs (GPIOs), two one-wire interface (OWI) connectors with separate data lines, and two two-wire interface (TWI) connectors (i.e., I2C). The ASN(x) can be easily programmed via a 6-pin AVR in-system programmer (ISP) connector. Additionally, for timestamping purposes or to have an external wake-up source for the MCU, a PCR85263A low-power RTC is available on the sensor node. Nevertheless, one of the most important features of the ASN(x) are the self-check measures added to the design to allow node-level fault diagnosis (see Section 4.5). The particular units, their components, and their characteristics are described in the following in detail.

### 4.1. Processing Unit

The core of the ASN(x) forms the ATmega1284P, a high-performance 8-bit AVR RISC-based MCU with rich on-chip peripherals. It has 128 kB ISP flash memory, 16 kB static random-access memory (SRAM), and 4 kB electrically erasable programmable read-only memory (EEPROM). The ATmega1284P can be clocked either by the internal 8 MHz RC oscillator or using an external clock source with up to 20 MHz. In the ASN(x), the MCU is clocked via an external 4 MHz crystal oscillator. As the MCU can execute most instructions in a single clock cycle, the processor runs almost 4 million instructions per second (MIPS). The ATmega1284P’s operational temperature range is −40 up to +85 ${}^{\circ }$C which makes it usable for indoor and (most) outdoor applications. Additionally, it offers many on-chip peripherals such as a 10-bit ADC with eight input channels, 8- and 16-bit timers, and several communication interfaces (i.e., two USARTs, one SPI, and one TWI) while requiring only a minimal amount of external (passive) components. We found that having two USART interfaces is beneficial for sensor nodes as one is often used to communicate with the radio transceiver and a second one is helpful when developing and debugging the node’s software. Also, the ASN(x) has two user LEDs that can be physically disconnected if not needed to save energy.

To upload the node software onto the MCU the 6-pin AVR ISP connector can be used. It is connected to the MCU’s SPI and allows to write data to the ISP flash memory. However, a programmer to connect the ASN(x) with the host computer is needed which is available for around $20. Alternatively, a bootloader can be used that allows uploading new programs via USART like it is done with most Arduino boards. Such a bootloader would occupy a certain amount of flash memory (e.g., 500 bytes in case of an *optiboot*-based bootloader) but would allow to easily update the sensor node’s software with just a serial connection (e.g., via an FTDI USB-to-serial adapter). Currently, we do not provide such a bootloader for the ASN(x), but we plan to develop one soon. For more information on *optiboot*, we refer an interested reader to https://github.com/Optiboot/optiboot (last accessed on 12 October 2021).

One of the most important characteristics of an MCU to be used in wireless sensor nodes is its power consumption and the availability of suitable power saving modes (i.e., sleep modes). The ATmega1284P provides six different software-selectable power saving modes with different clock domains remaining active and different wake-up sources for the MCU. In the most power-saving mode, the power-down mode, the external oscillator is stopped; only the watchdog timer (WDT) (if enabled) continues to operate. Since almost the entire MCU core is disabled only an external event such as an external interrupt, an TWI address match, or a reset (either external, brown-out, or initiated by the WDT) can wake the MCU up from this mode. The power consumption of the MCU can be further decreased by deactivating the WDT and the brown-out detector. Additionally, the power consumption is affected by the external (passive) wiring.

As sensor nodes in environmental monitoring applications are usually active for a very short time and spend the rest of the time in a sleeping state, a reliable wake-up source allowing for intervals in the granularity of minutes up to a few hours is needed. This is normally realized by an external RTC that generates an external interrupt for the MCU after a defined period. For the ASN(x), we included a PCF85263A low-power RTC that can be either operated as a calendar-optimized clock or as a stopwatch (i.e., an elapsed time counter). To generate a periodic wake-up signal (i.e., external interrupt) the stop-watch mode is most suitable where the desired interval can be easily configured. The PCF85263A is clocked by an external 32.768 kHz quartz crystal. However, it is of utmost importance to ensure that the interrupt generated by the RTC reliably wakes up the MCU (i.e., proper RTC and MCU configuration). Otherwise, the node may end up in a state where it never wakes up from the power-down mode again.

### 4.2. Sensing Unit

The ASN(x) has an onboard TMP275 low-power temperature sensor connected via TWI/I2C. It enables temperature measurements for ambient temperatures between −40 and +125 ${}^{\circ }$C with an accuracy of ±1 ${}^{\circ }$C over the full range and ±0.5 ${}^{\circ }$C for temperatures between −20 and +100 ${}^{\circ }$C, respectively. The conversion resolution can be configured in software between 9-bit (0.5 ${}^{\circ }$C granularity with 27.5 ms typical conversion time) and 12-bit (0.0625 ${}^{\circ }$C granularity with 220 ms typical conversion time). Additionally, it can be configured for a one-shot temperature measurement mode where the sensor performs one conversion on demand and remains in a low-power state for the rest of the time.

Since the ASN(x) is meant to be a generic platform for monitoring applications, however, the sensor node provides interfaces for various types of sensors rather than having several sensors mounted on the PCB. Thereby, the costs are kept to a minimum as no unused sensors are included, and similarly, the power consumption is not burdened by mounted but unneeded sensors. Depending on the application, the sensors required can be connected to the available pin headers offering GPIOs (9×), ADC inputs (6×) as well as digital interfaces such as USART (1×), SPI (1×), OWI (2×), and TWI (2×). To connect the sensors either cables connected to the pin headers can be used or a sensor add-on can be developed (an ASN(x) add-on template is provided at https://github.com/DoWiD-wsn/asnx_addon_template). The latter is beneficial if numerous nodes with the same set of sensors have to be deployed.

Also, some of the self-diagnostic measures (i.e., fault indicators) are sensorial. However, since their main purpose is node-level fault detection rather than actual sensor value monitoring/reporting, they will be discussed in Section 4.5.

### 4.3. Power Unit

As shown in Table 1, most of the available sensor nodes are directly powered by (two AA) batteries or use linear regulators. Directly supplying the sensor node does not need any additional hardware for voltage regulation which saves costs and does not add any additional power dissipation. However, this option entails the hazard of undervolting of the components by a depleting battery that can result in serious soft faults [7]. This threat is removed by using a linear regulator to ensure a stable supply voltage at the cost of bad energy efficiency as those regulators convert the voltage surplus to heat. Additionally, linear regulators only work as long as the input (battery) voltage is higher than the desired supply voltage. In case the sensor node is supplied with two AA batteries resulting in a nominal voltage of 3 V, a supply voltage of 3.3 V can not be realized with a linear regulator.

For these reasons, we employed a single inductor buck-boost DC/DC converter with a fixed output voltage of 3.3 V in our ASN(x), more specifically, the TPS63031. Due to its buck-boost capability, a wide input voltage range of 1.8 to 5.5 V is supported with an input-to-output efficiency of above 65%. Thus, the input voltage range perfectly fits the voltage range of two AA batteries/accumulators but also offers the possibility to attach other forms of energy sources as long as they do not exceed 5.5 V.

The energy efficiency of the TPS63031 mainly depends on two factors, namely the input voltage, and the output current. Regarding the former, the DC/DC converter operates more efficiently in buck mode, that is, in cases where the input voltage is higher than the output voltage. When supplied with two AA batteries the TPS63031 is operated in boost mode that offers a slightly worse efficiency that is, however, still above 65% and, thus, much better than a linear regulator-based solution.

Concerning the output current, the TPS63031 can provide up to 500 mA in boost mode and even up to 800 mA in buck mode when operated in normal operation mode. Such high values are rarely needed on sensor nodes. For output currents below 100 mA, the DC/DC converter offers a power-save mode. In this mode, the converter is operated asynchronously and stops whenever the output voltage is at or above its nominal value. Only in cases where the output voltage drops below its nominal value, the converter is started and ramps up the output voltage for one or several pulses. The efficiency of the converter over the full battery-powered supply range and the effects of the power-save mode are analyzed in Section 6.1.

### 4.4. Transceiver Unit

The ASN(x) has a 20-pin socket with a pin assignment commonly used in XBee through-hole technology (THT) modules whereby not all signals are connected to the MCU. In the current version of the ASN(x), the USART and the SPI signals are connected as well as the two pins responsible for controlling the pin-sleep functionality, that are, the sleep request and the sleep indication pins.

Initially, the ASN(x) was designed for using a Digi XBee 3 RF module, hence, the “(x)” in the name ASN(x). The module supports different networking protocols (i.e., Zigbee, IEEE 802.15.4, and DigiMesh). For each of these protocols, a corresponding firmware is provided by Digi. For the ASN(x), we currently use the Zigbee firmware to establish a Zigbee 3.0 network. The Xbee 3 module additionally has a BLE interface that can be activated for debugging or configuration purposes.

However, the 20-pin footprint and pin layout of the XBee modules have become a standard design. Today, numerous modules featuring different RF technologies are available in the “XBee” (or sometimes simply “Bee”) layout such as the Core2530 module with a TI CC2530F256 Zigbee radio (for more information on the Core2530 module, we refer to https://www.waveshare.com/wiki/Core2530_(B), accessed on 12 October 2021). For this reason, the “(x)” in ASN(x) may also be seen as an abbreviation for “eXtensible” as the sensor node easily extendable with any radio transceiver available in the XBee layout. The same concept is also used, for example, in the Libelium Waspmote to support a wide range of different applications and use cases with a single hardware platform ([78]).

### 4.5. Node-Level Indicators

To the best of our knowledge, the ASN(x) is the first sensor node that enables active node-level reliability by including so-called *fault indicators*. Fault indicators are enhanced self-diagnostic measures added to the sensor node design, both in hardware and in software (for more general information on fault indicators we refer to [4]). The diagnostic information obtained from the fault indicators allows us to infer qualitative information on the sensor node’s state of operation, or in other words, they indicate circumstances that facilitate erroneous behavior. These simple self-checks offer a good way to enhance the node’s reliability while requiring a minimal energy overhead only. Sensor data reported in times of active fault indicators are more likely to be corrupted (or even arbitrary) than data acquired in phases of dormant fault indicators.

We denote our fault indicators with the Greek letter χ (small chi) due to the name similarity of our fault indicators with indicator functions used in mathematics that are commonly represented with the letter χ. In the current version of the ASN(x), eight different fault indicators are implemented as listed in Table 2. Thereby, each indicator targets a different component or operational aspect of the sensor node as will be described in the following. These diagnostic data can either be checked on the sensor node and/or be sent to other network participants to establish a distributed fault-detection scheme. As presented in [4], the fault indicators can be categorized based on whether they are inherently available on the node (i.e., software metrics) or can be artificially added (i.e., additional hardware).

#### 4.5.1. Node Temperature Monitor

The first fault indicator is derived from temperature measurements of the ASN(x)’ components, in particular, the MCU’s surface temperature, the board temperature, and the radio transceiver core temperature. It is denoted as $\chi_{NT}$ where “NT” stands for “node temperature”.

The MCU’s surface temperature ($T_{MCU}$) is measured via a 103JT-025 thin-film negative temperature coefficient (NTC) thermistor affixed to the MCU’s surface. To get the respective temperature, the thermistor is used in a voltage divider in combination with a 10 kΩ balance resistor placed on the high side (i.e., connected to the supply voltage) while the thermistor is located on the low side (i.e., connected to ground). The voltage divider midpoint is connected to the MCU’s ADC. In order not to waste energy, the voltage divider can be enabled and disabled via an N-channel metal-oxide-semiconductor field-effect transistor (MOSFET) controlled by a GPIO. We use the Steinhart-Hart equation to calculate an approximation of the thermistor’s temperature based on the ADC’s conversion result and the thermistor’s characteristics (i.e., beta value).

The board temperature ($T_{BRD}$) is provided by the on-board TMP275 temperature sensor connected to the MCU via TWI. In our setup, we configured the sensor to provide us with temperature measurements with a 10-bit resolution (0.25 ${}^{\circ }$C granularity) that take approximately 55 ms for single conversions and, thus, having a good balance between measurement accuracy and conversion time required.

The radio transceiver core temperature ($T_{TRX}$) of the XBee 3 module is available as part of the diagnostic information provided by the module. It can be read from the module using AT commands (i.e., Hayes commands) issued via the USART interface.

As all three temperature measurements are taken from places on the sensor node and, thus, inside the node’s housing, all three measurements should be similar. While the absolute value may differ (i.e., have an offset of a few degrees Celsius), the trends of the temperature measurements should have a negligibly small difference as we expect the measurements to equally react to external influences. Therefore, the node temperature monitor fault indicator $\chi_{NT}$ is defined as the standard deviation of the changes of the three temperature measurements considering two consecutive measurements denoted as $\Delta T_{MCU}$, $\Delta T_{BRD}$, and $\Delta T_{TRX}$, respectively. $\chi_{NT}$ is calculated with:

(1) $$\chi_{NT}=\sqrt{\frac{{\left(\Delta T_{MCU}-\mu_{NT}\right)}^{2}+{\left(\Delta T_{BRD}-\mu_{NT}\right)}^{2}+{\left(\Delta T_{TRX}-\mu_{NT}\right)}^{2}}{3}}$$

where $\mu_{NT}$ is the mean value of the temperature readings calculated as:

(2) $$\mu_{NT}=\frac{\Delta T_{MCU}+\Delta T_{BRD}+\Delta T_{TRX}}{3}\;.$$

Thereby, a higher value of $\chi_{NT}$ indicates a higher probability of experiencing a faulty system condition. So far, we use a uniform weighting of the single temperature measurements. However, a future analysis may suggest using different weights.

Regarding the fault indicator categorization, the node temperature monitor implemented on the ASN(x) requires additional hardware (i.e., TMP275 temperature sensor, thermistor circuit) that can be, however, added to almost every sensor node. For this reason, this fault indicator belongs to the artificial generic indicators.

Some MCUs such as the ATmega328P have a core temperature sensor implemented into their ADC peripheral and, thus, would allow acquiring the MCU core temperature without the need of additional hardware. In such a case, a simpler $\chi_{NT}$ could be implemented as an inherent component-specific indicator by considering the MCU and radio core temperatures only.

#### 4.5.2. Supply Voltage Monitor

Previous analysis [4] has shown that aside from the ambient temperature especially the supply voltage of the sensor node has a significant influence on its proper operation. Consequently, our second fault indicator $\chi_{VS}$ considers the supply voltage on the sensor node measured by the MCU and the XBee independently.

The supply voltage level of the MCU can be measured without the need for additional hardware or circuitry using the on-chip ADC as described in Microchip’s application note AN2447 [98]. Similar to the temperature, also the supply voltage level of the XBee is provided as a diagnostic value retrievable via an AT command. For this reason, $\chi_{VS}$ is an inherent component-specific indicator as the measurements are inherently available, but are specific to the hardware components used.

The supply voltage is regulated by the on-board DC/DC converter and, in a fault-free operation, should be constantly 3.3 V (with minor fluctuations). We derive $\chi_{VS}$ as the absolute difference between the measured MCU supply voltage ($V_{MCU}$) and the radio transceiver supply voltage ($V_{TRX}$) with:

(3) $$\chi_{VS}=\left|V_{MCU}-V_{TRX}\right|$$

where the probability of a faulty condition is directly proportional to the value of $\chi_{VS}$.

#### 4.5.3. Battery Voltage Monitor

Aside from the supply voltage also the battery voltage offers vital information on the node’s state of operation. Thereby, especially the deviation between several consecutive measurements and the rate of change are important characteristics. To measure the battery voltage, we added a voltage divider consisting of two 10 kΩ resistors between the battery input voltage (before the DC/DC converter) and ground level. The midpoint of the voltage divider is connected to the MCU’s ADC. As two equal resistor values are used, the highest voltage level of the midpoint equals

(4) $$V_{ADC,max}=V_{BAT,max}\cdot \frac{R_{2}}{R_{1}+R_{2}}=\frac{V_{BAT,max}}{2}=2.75\;V$$

and, thus, stays below the maximum ADC input voltage of 3.3 V as long as the battery voltage does not exceed the maximum of 5.5 V. Due to the voltage divider ratio the voltage level applied to the ADC is half the level of the battery voltage. Therefore, the corresponding battery voltage can be calculated with:

(5) $$V_{BAT}=V_{ADC}\cdot \frac{2\cdot V_{VS}}{ADC_{max}}$$

where $V_{VS}$ is the supply voltage level (i.e., 3.3 V) and $ADC_{max}$ is the maximum conversion result depending on the ADC’s resolution (1023 in case of a 10-bit resolution). The voltage divider can be also be enabled/disabled via an N-channel MOSFET.

We defined the battery voltage monitor fault indicator $\chi_{BAT}$ to be the standard deviation of *N* consecutive measurements of the battery voltage as:

(6) $$\chi_{BAT}=\sqrt{\frac{1}{N}\sum_{i=1}^{N}{\left(V_{BAT,i}-\mu_{BAT}\right)}^{2}}$$

where $\mu_{BAT}$ is the mean value of the measurements calculated as:

(7) $$\mu_{BAT}=\frac{1}{N}\sum_{i=1}^{N}V_{BAT,i}\;.$$

A larger value of $\chi_{BAT}$ represents high deviations between consecutive measurements and, therefore, indicates possibly erroneous circumstances.

For the battery voltage monitor, an additional voltage divider to measure the battery voltage is used that can, however, be added to almost every sensor node. Therefore, this indicator counts as an artificial generic indicator.

#### 4.5.4. Active Runtime Monitor

The active runtime fault indicator monitors the length of the period the sensor node is active. The active phase follows a pre-defined sequential processing of certain tasks and should, therefore, be of constant length in every iteration. Significant deviations in the length of the active phase can indicate possibly erroneous circumstances.

In the current version of the ASN(x), the active runtime monitor indicator $\chi_{ART}$ is realized using the 16-bit timer1 peripheral of the MCU. The timer is started as soon as the node wakes up and stopped shortly before entering power-down mode. The counter value after stopping the timer is directly proportional to the length of the active phase. In our implementation, we configured the timer module to run with a prescaler of 1024 resulting in a tick length of 256 μs for a clock frequency of 4 MHz. The time spent in the active phase equals the counter value multiplied by the length of a tick. Therefore, the measurable time interval of the 16-bit timer is [256 μs, 16.78 s]. If other time intervals (e.g., shorter or longer) are needed, the timer’s prescaler needs to be adjusted.

As we expect the period of the active phase to be of more or less constant length, we define $\chi_{ART}$ as the standard deviation of *N* consecutive measurements (measured in milliseconds). Thereby, we consider the magnitude of the difference rather than the absolute values, hence, we calculate $\chi_{ART}$ as the common logarithm of the standard deviation with:

(8) $$\chi_{ART}=\log_{10}\left(\sqrt{\frac{1}{N}\sum_{i=1}^{N}{\left(t_{active,i}-\mu_{ART}\right)}^{2}}\right)$$

where $t_{active,i}$ is the length of the *i*-th measurement and $\mu_{ART}$ is the mean value of the measurements calculated as:

(9) $$\mu_{ART}=\frac{1}{N}\sum_{i=1}^{N}t_{active,i}\;.$$

To avoid negative values of $\chi_{ART}$, the logarithm is only calculated in case the standard deviation is greater than one. In case the standard deviation is smaller or equal to one, $\mu_{ART}$ is defined to be zero as the difference is negligibly small. Again, a larger value refers to a higher probability of abnormal circumstances possibly caused by faults. In our implementation, we used five consecutive values (N=5) for the evaluation of $\chi_{AT}$. However, further analysis on the optimal number of measurements would be beneficial to increase the indicator’s expressiveness.

As only on-chip resources of the MCU are used, $\chi_{ART}$ refers to an inherent component-specific indicator. It could be argued that it is an inherent common indicator as almost all MCUs have timer modules, however, it still depends on the MCU and, thus, is component-specific.

#### 4.5.5. Reset Monitor

A node reset is an action normally taken by the hardware or software in situations where proper operation can not be continued anymore (such as a watchdog reset). Therefore, a node reset is a clear sign of an unsafe operational condition often originating from faults. While the node may continue its proper operation after a reset, the probability of faulty circumstances is higher after a reset especially if several resets happen during a short period. Additionally, the reason for the reset is relevant in deciding how probable faulty conditions are.

As a consequence, we implemented a reset monitor indicator $\chi_{RST}$ that is based on the number of resets happening in a certain timespan and the sources of the resets (e.g., the MCU module causing the reset). Thereby we leverage the 8-bit MCU status register (MCUSR) available on most AVR MCUs. It provides information on which source caused the latest reset. The available sources indicated by corresponding flags in the MCUSR are:

- bit 0: power-on reset,
- bit 1: external reset (via the reset pin),
- bit 2: brown-out reset (in case the brown-out detection is enabled), and
- bit 3: watchdog reset.

We defined that the probability of faults is higher after a watchdog reset than after a power-on reset. Correspondingly, we use the bit position of the flags to weigh the reset sources where a higher weight refers to a higher probability of impaired operation. The ATmega1284P also has a flag for resets caused by the Joint Test Action Group (JTAG) interface (bit 4), but as we do not use JTAG we ignored it. Bits 5 to 7 are not used and always read as zero. However, the MCUSR needs to be cleared manually to detect whether new resets have happened since since its last access.

Aside from the reset source, also the amount of resets during a certain period is considered. For this reason, we implemented $\chi_{RST}$ as a function based on its previous value, the current value of the MCUSR, and a time-dependent exponential decay with a pre-defined rate. In each iteration (active phase), the value of $\chi_{RST}$ is updated using the following equation:

(10) $$\chi_{RST,n}=\chi_{RST,n-1}\cdot \lambda_{RST}+\mathrm{MCUSR}$$

where $\lambda_{RST}$ is the inverse decay rate and the initial value $\chi_{RST,0}=0$. In our implementation, we defined $\lambda_{RST}$ as 0.92 resulting in a decay of 8% of the previous value per iteration. However, to keep the value of $\chi_{RST}$ during a reset, its value is written to the EEPROM. Since the EEPROM has a limited number of write cycles, the value is only updated in the EEPROM if the current value differs from the previous one.

The $\chi_{RST}$ is categorized as an inherent component-specific indicator as it requires the availability of EEPROM and reset-related information (such as the MCUSR in case of AVR MCUs), but does not need additional hardware or circuitry.

#### 4.5.6. Software Incident Counter

To monitor the stability of the software execution, we defined an incident counter indicator $\chi_{IC}$ that collects information on how many software function calls failed during a certain period. This counter, however, relies on the availability of corresponding function return values expressing whether the function call was successful or failed. For this reason, we implemented the majority of our software modules in a way that they provide such information, that is, if the processing was successful a *pass* is returned. In case of problems (e.g., function timeouts, incorrect responses, failed value checks) the functions return *fail*.

Based on these return values, we defined $\chi_{IC}$ as

(11) $$\chi_{IC,n}=\left\{\begin{array}{cc} \chi_{IC,n-1}+1, & \mathrm{if}\;a\;\mathrm{function}\;\mathrm{returned}\;\mathrm{with}\;\mathrm{fail}\\ \chi_{IC,n-1}-1, & \mathrm{if}\;\mathrm{pass}\;\mathrm{AND}\;\chi_{IC,n-1}>0\\ 0, & \mathrm{otherwise} \end{array}\right.$$

where the initial value is $\chi_{IC,0}=0$. Thereby, a high counter value indicates a rather unsafe state of operation in which faults are more likely to occur. However, if $\chi_{IC}$ exceeds a predefined threshold, the sensor node is reset.

As the incident counter indicator $\chi_{IC}$ does not require any additional hardware and has no dependency on the components used, it belongs to the inherent common indicators that can be added to any sensor node.

#### 4.5.7. ADC Self-Check

Our previous research [7] revealed that especially the MCU’s ADC and USART modules are susceptible to faults caused by fluctuations in the supply voltage and/or temperature. These modules, however, are essential for the correct operation of the sensor node and, thus, checking their proper function during runtime is crucial.

To check the ADC module’s operation, we added a voltage divider with a fixed ratio to the ASN(x) design. The voltage divider is placed between the supply rail and the ground potential. Its midpoint is connected to the MCU’s ADC. As the ratio of the voltage divider is fixed, the ADC’s conversion result of the respective channel should be stable (aside from minimal conversion-related fluctuations).

In the current version of the ASN(x), two 10 kΩ resistors are used. Again, the voltage divider used can be enabled/disabled via an N-channel MOSFET to save energy. The voltage level at the ADC input depends on the resistor values ($R_{1}=R_{2}=10\;k\Omega$) and equals:

(12) $$V_{ADC,CH0}=V_{VS}\cdot \frac{R_{2}}{R_{1}+R_{2}}=\frac{V_{VS}}{2}\;.$$

The expected conversion result of the ADC (10-bit resolution and the analog reference voltage $V_{AREF}$ connected to $V_{VS}$) equals:

(13) $$ADC_{expected}=\frac{ADC_{max}}{V_{AREF}}\cdot V_{ADC}=\frac{2^{10}-1}{V_{VS}}\cdot \frac{V_{VS}}{2}\approx 511\;.$$

The ADC self-check indicator $\chi_{ADC}$ is now defined as the deviation of the actual conversion result from the expected value and is calculated with:

(14) $$\chi_{ADC}=\left|ADC-ADC_{expected}\right|$$

where larger values indicate faulty circumstances. Since this indicator requires an additional voltage divider that can, however, be added to any sensor node with an ADC, it is an artificial generic indicator.

#### 4.5.8. USART Self-Check

Similarly to the ADC self-check, we also implemented a way to evaluate the USART’s operation for correctness. Thereby, we leverage the availability of two USART modules in the ATmega1284P, named USART0 and USART1. USART0 is used for communication with the XBee radio module and USART1 can be used for debugging purposes during the development phase. After deployment, the USART1 can be used to monitor the USART0 interface employing a loop-back test. To do so, the ASN(x) has two open solder jumpers that can be bridged to form a loop. After that, every time data is sent via USART0 the same data should arrive at USART1. Consequently, the USART self-check indicator $\chi_{USART}$ is defined as:

(15) $$\chi_{USART}=\sum_{i=1}^{\left|\right|D_{TX}\left|\right|}\Delta_{TXRX,i}$$

with

(16) $$\Delta_{TXRX,i}=\left\{\begin{array}{cc} 1, & \mathrm{if}\;D_{RX,i}\neq D_{TX,i}\\ 0, & \mathrm{otherwise} \end{array}\right.$$

where $D_{RX}$ refers to the array of bytes received by USART1 and $D_{TX}$ refers to the array of bytes transmitted by USART0, both regarding the data of one message transmission. Thus, it expresses the number of bytes that have not been correctly received by the loopback interface (i.e., USART1).

The implementation of $\chi_{USART}$ requires the availability of two USART interfaces (component-specific) and an external connection between both (loop-back; additionally added). Therefore, $\chi_{USART}$ counts as an artificial component-specific indicator.

## 5. Evaluation Experiment Setup

In the following, we present the practical evaluations used to show that the fault indicators enhance the reliability of the nodes by improving the detection rate of sensor node faults while posing only a negligibly small energy overhead to not diminish the energy efficiency.

The analysis of (soft) faults in WSN is difficult as many factors influence the node’s operation and can, either alone or in combination, lead to a faulty node behavior. Additionally, simulations are of no use in this context as most fault-related effects occur in real systems only. For this purpose, we performed practical experiments in three different settings to give our analysis a broader scope by covering as many operational and environmental circumstances as possible.

**Figure 10:**
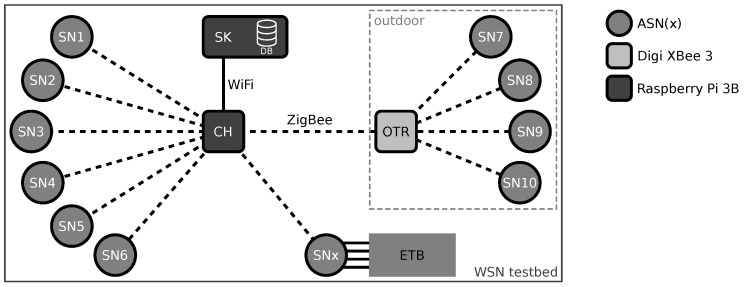
Architecture of the experiment setup (WSN testbed).

Our experiments were performed in a smart garden setting where four environmental parameters related to plant growth were monitored, namely ambient air temperature and relative humidity as well as soil temperature and moisture level. As shown in Figure 10, we deployed a WSN testbed consisting of several sensor nodes (SNs) in three different settings:

1. an indoor deployment consisting of six SNs,
2. an outdoor deployment consisting of four SNs, and
3. a lab experiment setup analyzing one dedicated SN controlled by our embedded testbench (ETB).

**Figure 11:**
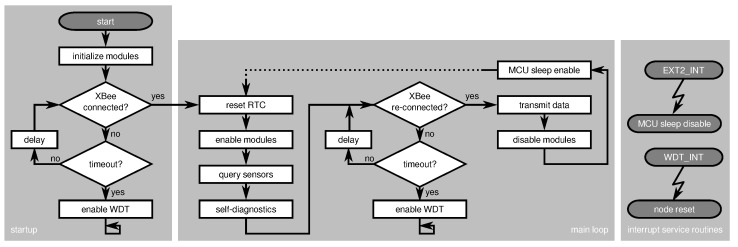
Simplified sensor node demo software flowchart.

The basic routine of all three setups is very similar. All sensor nodes were programmed in C-language on the bare metal (without an OS) following the procedure shown in Figure 11. The respective sources are available at https://github.com/DoWiD-wsn/avr-based_sensor_node/tree/master/source/004-sensor_node_demo. The sensor nodes monitor the aforementioned environmental parameters and the fault indicators presented in Section 4.5 where the raw information is forwarded every 10 min to the Raspberry Pi-based cluster head (CH) via Zigbee (CH setup information and the used Python script are available at https://github.com/DoWiD-wsn/RPi_cluster_head). The data is then further transmitted to the sink node (SK) via WiFi (also Raspberry Pi-based) where the data is finally stored in a structured query language (SQL) database. Information on the sink node (SK) and its setup can be found under https://github.com/DoWiD-wsn/RPi_sink_node. Currently, the assessment of the fault indicators is performed centrally on the SK.

All SNs are equipped with an XBee 3 radio (all XBee radios run the “*Digi XBee3 Zigbee 3.0 TH*” firmware version 100D) configured to transmit at the lowest power level (i.e., at −5 dBm) to decrease the overall power consumption of the node (the XBee 3 configuration used is available at https://github.com/DoWiD-wsn/avr-based_sensor_node/tree/master/source#configuration-for-asnx).

To ensure a reliable Zigbee network connection of the SNs placed outdoors, we additionally deployed an outdoor relay node (OTR) which consists of an XBee 3 module operated standalone in network router configuration that is supplied by a wired power supply. In contrast to the SNs, the XBee radios of the OTR and CH use the highest power level available, that is +8 dBm.

### 5.1. Indoor Deployment

The indoor deployment consists of six nodes (*SN1* to *SN6* in Figure 10) that are placed on top of plant pots in the living area of a residential property. Thereby, *SN1* and *SN2* were equipped with ambient temperature and relative humidity sensors (AM2302 sensors) while *SN3* to *SN6* were equipped with a temperature sensor to measure the soil temperature (DS18B20 sensors) and sensors to measure the soil’s moisture level (Adafruit STEMMA soil sensors). The indoor deployment ran for 150 days where each sensor node sent an update every 10 min.

With this deployment, we analyze the behavior of the ASN(x) including their fault indicators during a normal operation in a mostly controlled environment. In this environment, no extreme environmental disturbances such as high temperatures or strong rain compromised the nodes’ operation. Thus, the data acquired from the indoor deployment provide some kind of reference measurements, or in other words, how the sensor nodes behave in a stable environment.

### 5.2. Outdoor Deployment

Especially the harsh conditions posed by the environment of outdoor deployments have been shown to significantly impact the behavior of sensor nodes and the probability of node faults, respectively. For this reason, we deployed four sensor nodes (*SN7* to *SN10* in Figure 10) in different locations of raised beds planted with different crops. All four nodes were equipped with the same sensors as *SN3* to *SN6* (see Section 5.1), except for *SN7* which had an additional AM2302 ambient temperature and relative humidity sensor installed. The outdoor testbed was active during August and September 2021 where several weather extremes such as sudden heavy rain, strong winds, and significant temperature fluctuations occurred that, however, posed perfect conditions for our evaluation.

In contrast to the indoor deployment, the outdoor installation provided us with data from sensor nodes in normal operation but an uncontrolled and harsh environment. Thereby, especially direct sun radiation and heavy rainfalls posed challenging conditions for our ASN(x) where the latter also caused the leaking of water into the housing of some nodes resulting in partial short circuits. As the same sensors were used as in the indoor deployment, we were able to identify differences in the node/sensor behavior caused by the environmental influences (cf. Section 6.2).

### 5.3. Embedded Testbench (ETB)-Based Lab Experiments

**Figure 12:**
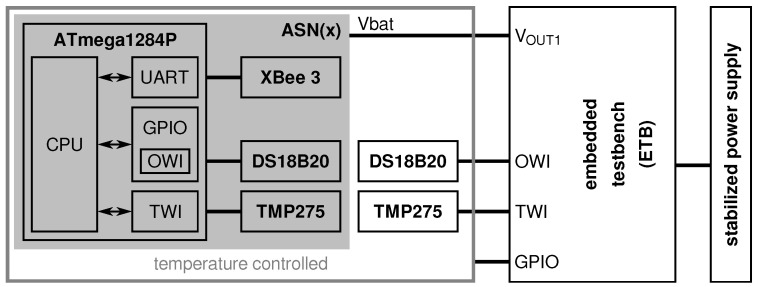
ETB-based lab experiment setup.

In addition to the indoor and outdoor deployments, we used a lab experiment setup (see Figure 12) to further investigate the effects of the supply voltage and ambient temperature (separate and in combination) on the sensor node’s operation. Additionally, we used this setup to analyze the ASN(x)’ power consumption and energy efficiency. The measurements of the ASN(x)’ power consumption were augmented with energy measurements provided by a Joulescope (see https://www.joulescope.com/, accessed on 12 October 2021) connected between the power supply and the sensor node as presented in Section 6.1.

As depicted in Figure 12, the lab experiment setup consists of a dedicated sensor node (*SNx* in Figure 10) and the *Raspberry Pi 3*-based embedded testbench (ETB) acting as an experiment controller. Information on the ETB as well as its design files and Python sources are available at https://github.com/DoWiD-wsn/embedded_testbench.

In this setup, the ASN(x) is equipped with a DS18B20 in addition to the onboard TMP275 temperature sensor. As shown in Figure 12, both sensors are duplicated with one set connected to the ASN(x) and the second connected to the ETB for reference measurements. Using the reference measurements, we can identify sensor data that is corrupted due to node-level effects.

**Figure 13:**
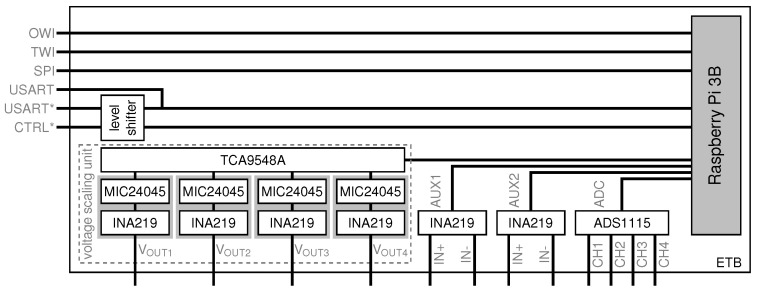
Basic components of the embedded testbench (ETB).

The ETB encompasses a Raspberry Pi add-on and Python sources to enable the testing, analyzing, and profiling of embedded systems with a focus on low-power devices. As shown in Figure 13, it offers four independent power outputs each equipped with a wattmeter, two auxiliary wattmeters, a four-channel 16-bit ADC, and connectors for various communication interfaces. Each power output consists of a MIC24045 buck converter with a programmable output voltage between 0.64 V and 5.25 V. Using this *voltage scaling unit*, we can precisely adjust the ASN(x)’s supply voltage to mimic the effects of a depleting battery or other effects such as temporary voltage fluctuations (e.g., caused by short circuits). Additionally, the ETB provides four signals dedicated to low-level experiment control and data exchange with the device under test (DUT). These test control signals and the USART interface have MOSFET-based bi-directional level shifters to prevent effects caused by different voltages of the logic levels.

In our lab experiment setup, we can also vary the ambient temperature using a 100 W infrared lamp in combination with a modified hair dryer (both controlled via a relay card connected to GPIOs of the ETB). During our experiments, we supplied the ASN(x) with voltages between 0 and 5 V and exposed the node to temperatures up to +70 ${}^{\circ }$C. Due to the adjustable environmental parameters, the lab experiment setup allowed us to analyze the ASN(x)’ behavior during an impaired operation in a controlled environment. However, as the ETB controls the sensor node supply voltage and ambient temperature, our experiments can be automated using Python scripts, hence, the experiments are reproducible to better distinguish between sporadic and recurring effects.

In contrast to the sensor nodes used in the indoor and outdoor deployments, the *SNx* was configured to send updates every minute to have data of finer granularity. Additionally, the ETB kept track of the node’s supply voltage and current consumption (via the *voltage scaling unit*) as well as its reference measurements. The result of our experiments and the findings of both deployments are discussed in Section 6.

## 6. Results

In this section, the results of our experiments (cf. Section 5) are presented and the corresponding findings are discussed. As mentioned before, two characteristics of sensor nodes are of paramount importance, namely:

- reliability and
- energy efficiency.

Only if both are satisfied, the sensor nodes can provide data of high quality over the long lifetimes usually expected from WSNs. Therefore, we will have a look at the result of the ASN(x)’ power consumption and energy efficiency measurements in Section 6.1 followed by an analysis of the implemented fault indicators and their suitability in indicating a faulty node operation in Section 6.2. Regarding the latter, we separately show that:

- the fault indicators can indicate an impaired node operation.
- the fault indicators do not cause false alarms in case of rare but proper events.
- some types of faults were not detected by our current fault indicators.

### 6.1. Power Consumption

To analyze the ASN(x)’ power consumption, we put the on-board components into specific states and measured the power consumed in each state. The used source code can be found at https://github.com/DoWiD-wsn/avr-based_sensor_node/tree/diagnostics/source/005-power_consumption.

During these measurements, no external sensors were connected to the ASN(x). We took 500 measurements for each state and calculated the mean value. The current and the corresponding power consumption at 3.3 V supply voltage in the particular states are:

- 13.4 mA/44.22 mW (MCU idling; XBee enabled; diagnostics enabled),
- 12.2 mA/40.26 mW (MCU idling; XBee enabled; diagnostics disabled),
- 4.68 mA/15.44 mW (MCU idling; XBee disabled; diagnostics disabled).
- 36.7 μA/121.11 μW (MCU power-down; XBee disabled; diagnostics disabled)

**Figure 14:**
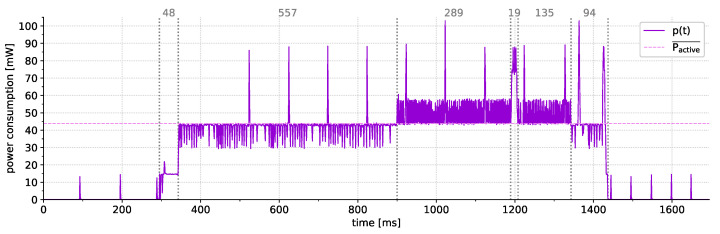
Current consumption in and duration of the active phase.

The power consumption was further analyzed using the aforementioned Joulescope connected in series to the voltage supply. However, for these measurements, the ASN(x) was equipped with an AM2302 ambient temperature and relative humidity sensor to better reflect the consumption of the sensor node in a real application setting. With the sensor node demo software depicted in Figure 11, the power consumption measured by the Joulescope is shown in Figure 14 where the Joulescope was configured to measure the current with a sampling frequency of 2 MHz and a granularity of the respective current measurements of 1.5 nA. The visible spikes are caused by the TPS63031 DC/DC converter running in power-saving mode as described in Section 4.3.

In Figure 14, also the particular states of the sensor node and their duration are visible. It takes about 48 ms for the CPU to become active after receiving the wake-up signal (i.e., external interrupt from the RTC), requesting the XBee to wake-up, and the XBee to be ready for operation ($\bar{I_{S1}}=4.68\;\mathrm{mA}$). For about 557 ms the ASN(x) is querying the attached sensors and deriving certain self-diagnostic metrics ($\bar{I_{S2}}=13.4\;\mathrm{mA}$). This phase, however, takes the longest time and is partly caused by a delay between the XBee’s wake-up and the Zigbee network rejoin (cf. Section 3.2.1). The transmission of data from the MCU to the XBee module via the USART interface (at 9600 baud) takes approximately 289 ms ($\bar{I_{S3}}=15.7\;\mathrm{mA}$) while the actual transmission via Zigbee only takes around 19 ms ($\bar{I_{S4}}=24.48\;\mathrm{mA}$). In the following 135 ms the XBee module waits for the message recipient to acknowledge the transmission and reports the corresponding return value back to the MCU ($\bar{I_{S5}}=14.27\;\mathrm{mA}$). For the next 94 ms, the ASN(x) finishes its processing of data and requests the XBee module to go back to sleep mode ($\bar{I_{S6}}=13.4\;\mathrm{mA}$). Overall, in the present demo case the ASN(x) spends about 1142 ms in one of the active states and is put to the power-down state the rest of the time ($\bar{I_{S7}}=36.7\;\mu A$).

The energy consumed by the ASN(x) in one 10 min interval is the cumulative sum of the energy consumed in each state and equals:

(17) $$Q_{node,10min}=\sum_{i=1}^{\left||S|\right|}\left(\bar{I_{Si}}\cdot t_{Si}\right)=37.86\;\mathrm{mAs}\equiv 10.52\;\mu \mathrm{Ah}$$

where *S* is the set of states with their respective length and current consumption as presented above.

In our setup, the sensor nodes were powered by two Alkaline LR6 AA batteries ($Q_{bat}$ = 2600 mAh). Therefore, the expected battery life can be estimated as follows (a 10 min interval equals 6 updates per hour):

(18) $$t_{bat}=\frac{Q_{bat}}{Q_{node,10min}\cdot 6}\cdot t_{1h}=\frac{2600\;\mathrm{mAh}}{10.52\;\mu \mathrm{Ah}\cdot 6}\cdot 1\;h\approx 41191\;h\equiv 4.7\;\mathrm{years}$$

To confirm our estimation, we measured the energy consumed by the ASN(x) using the Joulescope for 6 h (again at a sampling frequency of 2 MHz) resulting in an average energy consumption of 65.1 μAh per hour (= 10.85 μAh per 10 min) which equals an expected battery life of 4.56 years.

Next, we analyzed the power efficiency η of the DC/DC converter used on the ASN(x). As described in Section 4.3, its power efficiency depends on the input voltage level and the output current. With the “*supply voltage sweep with plot*” example script of our ETB (see https://github.com/DoWiD-wsn/embedded_testbench/tree/master/source/examples), we analyzed the power efficiency of the TPS63031 by applying varying input voltages, measuring the input current and calculating the corresponding input power $p_{in}$. Thereby, voltages between 1.5 and 3.5 V were applied (in descending order) and 1000 measurements per voltage level with 2 ms between have been taken. During the measurements, the ASN(x) was in an idling state (for the source code, see https://github.com/DoWiD-wsn/avr-based_sensor_node/tree/diagnostics/source/006-idling). The mean average current consumption at each level has then been compared with a reference measurement $P_{ref}$ of a directly supplied ASN(x) (bypassing the TPS63031) at 3.3 V to calculate the converter efficiency.

**Figure 15:**
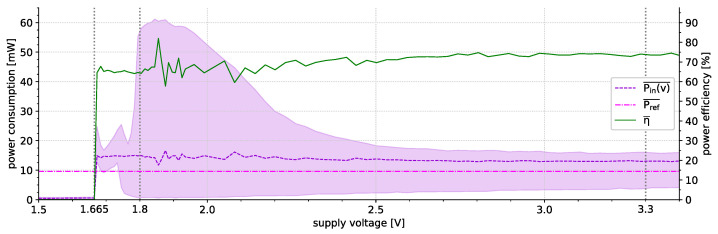
Average power efficiency of the DC/DC converter.

The result of this analysis is depicted in Figure 15 where the shaded area shows the span between the minimum and maximum values per voltage level. With lower input voltages the margin between minima and maxima gets larger. Between 1.665 and 1.8 V the TPS63031 consumes energy without providing an output voltage. With our measurements, we were able to confirm the high power efficiency of the TPS63031 of ≥65%.

Considering the results of the power consumption and energy efficiency analysis shown above, the ASN(x) provides an energy-efficient operation comparable to related sensor nodes (see Table 1 in Section 3.2) even though it includes self-diagnostic measures (i.e., fault indicators). Thus, the energy overhead required by the implemented indicators is negligibly small and, therefore, does not influence the expected battery life notably.

### 6.2. Indicator Evaluation

Currently, there are eight distinct fault indicators implemented in our ASN(x) as described in Section 4.5. These indicators aim at supporting fault detection on a node level by incorporating data able to infer information on the sensor node’s state of operation. Aside from improving the fault detection rate, these indicators especially help in distinguishing between the effects of faults on the reported sensor data and rare but proper events in the sensed physical phenomenon.

To show that our fault indicators indeed support the detection of faults and allow us to separate the effects of faults from proper events, we analyzed the data collected during our tripartite experiments. In total, more than 1.5 million data points were collected from both deployments (indoor and outdoor) and the lab experiment. These data points include the measurements of the sensors attached to the node and the self-diagnostic data collected on each sensor node. In the following, we show the expressiveness of the fault indicators by providing examples of:

- successfully detected faults (see Section 6.2.1),
- proper events that can be distinguished from faults (see Section 6.2.2), and
- faults that have not been indicated by our current fault indicators (see Section 6.2.3).

#### 6.2.1. Fault Indication

The basic working principle and related examples of the fault indicators have been presented in [4]. In addition, we present two examples of fault-induced sensor data anomalies successfully indicated by our implemented fault indicators. For better visibility, we applied feature scaling using min-max normalization on the fault indicator values shown in the following figures.)

**Figure 16:**
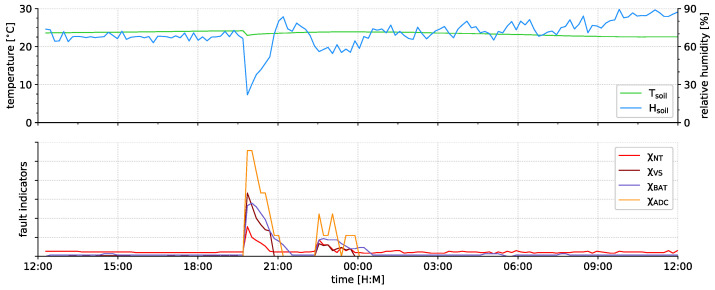
Faulty sensor reading successfully reported by the fault indicators.

The first example depicted in Figure 16 shows the sensor data (i.e., soil temperature and humidity) and selected fault indicator values of the sensor node *SN6* (indoor deployment) collected between 13 July 2021 and 14 July 2021.

There is a clear anomaly in the readings from the soil humidity sensor (STEMMA soil sensor) between 19:00 and 21:00 that was most probably due to temporary and partial short circuits caused by water dripping over the unprotected sensor node while watering the nearby plants. During the same period, several indicators reacted significantly. Additionally, the same indicators showed abnormal behavior between 22:00 and 00:00 indicating that during this time the node’s operation was either again or still impaired.

**Figure 17:**
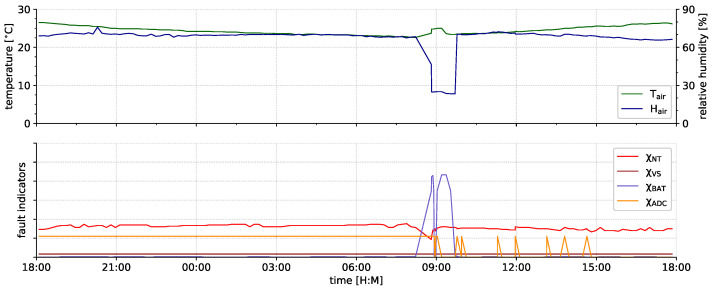
Fault-induced data anomaly highlighted by the fault indicators.

In the second example illustrated in Figure 17, the sensor data and indicator values of *SN1* (also indoor deployment) reported between 10 August 2021 and 11 August 2021 are shown. More specifically, the sensor data depicted are the ambient air temperature and relative humidity measurements provided by the attached AM2302 sensor. At around 09:00 the air humidity readings suddenly dropped significantly for a certain period (also the temperature readings showed some irregularities). At about the same time, the battery voltage monitor fault indicator showed a strong reaction suggesting a problem in the node’s voltage supply. The other supply voltage-related indicator (i.e., $\chi_{VS}$) did not show any irregularities. Only the node temperature monitor $\chi_{NT}$ reacted slightly indicating sudden changes in the diagnostic temperature measurements. However, the strength of the fault indicator concept stems from the combination of several indicators that, in combination, indicate possibly erroneous circumstances more reliably.

In addition to runtime fault indication, the implemented fault indicators were also helpful during the ASN(x) development to identify design bugs in the node software as well as manufacturing defects of the nodes such as bad solder connections. Thereby, we were also able to find more sophisticated software bugs such as a race condition between the interrupt-driven USART transmission and the decoupling ring-buffer implemented in software. Also, the reset indicator helped in identifying software bugs such as logic or off-by-one bugs causing infinite loops.

#### 6.2.2. Event Indication

**Figure 18:**
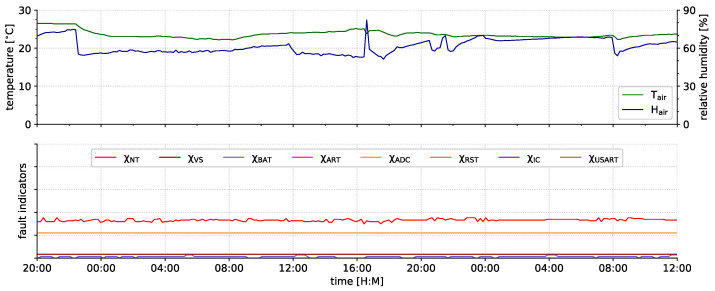
Example of a proper event distinguishable via the fault indicators.

Aside from improved fault detection, the fault indicators are especially useful in distinguishing between data anomalies caused by proper events from those caused by node faults. In this context, a data anomaly not causing the indicators to react is more likely due to an event rather than being fault induced. For example, Figure 18 shows the air temperature and humidity measurements and the fault indicator values reported by *SN1* (indoor) between 29 June 2021 and 1 July 2021. There are three events in the air humidity measurements, two abrupt changes (one on 29 June 2021 at 22:30 and a second on 1 July 2021 at 08:00) and one spike on 30 June 2021 at around 16:35.

In all three cases, there was no reaction of the fault indicators and, thus, no evidence that these anomalies were caused by sensor node faults. However, all three events can be attributed to changes in the environment caused by human actions, that are, in the cases of the abrupt changes the nearby outer door was left open to air the room, and in case of the spike, a nearby plant had a pest infestation and was treated with a spray bottle. Additionally, there are fluctuations in the relative air humidity on 30 June 2021 between 20:00 and 23:00 caused by cooking in an adjacent room.

Without additional information from the fault indicators, these events could have easily been misclassified as fault-related anomalies. Although neighboring nodes reacted to the open door and the cooking, too, this is especially true for the spike as no other node was affected by the sudden increase in humidity concentrated on a small area only.

#### 6.2.3. Undetected Faults

**Figure 19:**
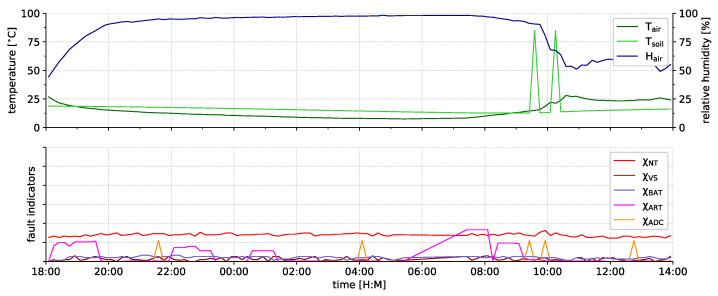
Example of a fault not highlighted by the fault indicators.

Nevertheless, some faults do not cause our (current) fault indicators to react. For example, the sensor node *SN7* used in the outdoor deployment reported incorrect values after heavy rainfall as shown in Figure 19 (data captured between 6 September 2021 and 7 September 2021). There are two times where the sensor node reported a temperature of 85 ${}^{\circ }$C while the outdoor temperature during this period never exceeded 25 ${}^{\circ }$C. Also, during this time there was no direct sunlight or any other reasonable explanation for these two deviations. Thus, we suppose that both spikes were caused by sensor faults due to humidity in the sensor’s wiring that did not cause any detectable symptoms on the sensor node (i.e., fault indicator reactions). Such outlier can, however, usually be easily detected as such large gradients are not possible in temperature curves in normal outdoor environments.

As can be seen in Figure 19, in contrast to the fault indicator values of the indoor nodes, some of the fault indicators showed notably more noise in the outdoor deployment although the same ASN(x) hardware and software was used. This, in turn, shows to what extent the environmental conditions of outdoor deployments impact the sensor nodes’ operation.

## 7. Conclusions

In this article, we have presented the *AVR-based Sensor Node with Xbee radio*, or short ASN(x), an open-source sensor node platform for monitoring applications such as environmental monitoring. The platform encompasses the node hardware (i.e., the sensor node) and the corresponding software components (i.e., software toolchain and libraries). It mainly uses low-power components to minimize power consumption and, thus, enable a long battery life. In contrast to related sensor nodes, the ASN(x) offers active node-level reliability based on the concept of fault indicators. With the help of these indicators, the detectability of node faults is improved and the distinction between sensor data anomalies caused by rare but proper events in the sensed phenomenon and fault-induced abnormalities is possible. This improves the WSN’s overall reliability with both, a long battery life of the sensor nodes and a high quality of the data acquired.

Using a tripartite practical setup consisting of an indoor (150 days with six nodes) and an outdoor (50 days with 4 nodes) deployment as well as a lab experiment we showed that the implemented fault indicators can indeed identify faulty sensor readings while not posing a burden to the node’s power consumption. As a result, the energy efficiency of the ASN(x) is comparable to related sensor nodes. For example, powered by two Alkaline AA batteries the ASN(x) can operate for more than 4 years with an update interval of 10 min. To show the efficiency of the fault indicator concept, we presented a selection of examples of how the indicators react to node faults and proper events. Also, based on the practical results we discussed the limitations of the indicator concept.

Currently, the evaluation of the fault indicators is performed centrally on a server with manual intervention. One of the next steps is to analyze the particular fault indicator to get information on their overall expressiveness, the types of faults they react to, and thresholds to be used for automated detection. Especially the latter is important to ensure reliable detection while keeping the number of false alarms low. We are also working towards a lightweight concept to evaluate the indicators on the node level. This would allow us to include the fault indicators in a fault detection approach as well as to further decrease the energy consumption by minimizing the communication overhead. In this context, the development of a distributed concept where the sensor nodes share information on the status with their neighbors will be an interesting advancement. Additionally, we will continue to find further fault indicators and practically evaluate those, that currently only have been analyzed theoretically (i.e., OS-related characteristics).

## Figures and Tables

**Table 1:** Overview of the characteristics of WSN nodes.

	Sensor Node	Year	MCU/SoC	Arch. [bit]	F_CPU_ [MHz]	Flash [kB]	RAM [kB]	EEPROM [kB]	Radio Transceiver	Communication Standard	V_core_ [V]	V_bat_ [V]	Active Mode [mW]	Power-Saving [μW]	Voltage Regulation	Energy-Efficiency	Self-Diagnostic	Open Source	Available	Price [$]
**Commercial**	UC Berkeley TelosB [73]	2005	MSP430F1611	16	8	1072 ^a^	10	16	CC2420	IEEE 802.15.4 (Zigbee)	3.0	1.8–3.6	6.6	20.1	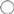	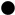	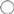	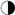	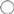	99.00
	ETH Zürich Btnode [74]	2005	ATmega128L	8	8	128	64 ^b^	4	ZV4002 + CC1000	IEEE 802.15.1 v1.2 + 433/868 MHz	3.3	0.5–4.4	39.6	9900	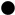	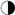	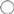	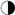	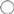	215.00
	UC Berkeley IRIS [75]	2007	ATmega1281	8	7.37	640 ^c^	8	4	AT RF230	IEEE 802.15.4 (Zigbee)	3.0	2.7–3.6	26.4	26.4	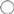	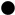	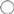	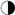	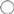	115.00
	SHIMMER [76]	2010	MSP430F1611	16	8	48	10	16	CC2420 + RN-41	IEEE 802.15.4 + 802.15.1 v2	3.3	1.8–3.6	5.9	16.8	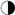	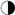	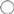	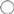	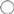	269.00
	OpenMote CC2538 [77]	2015	CC2538SF53 ^g^	32	32	512	32	–	CC2538 ^g^	IEEE 802.15.4 (6TiSCH)	3.3	2.0–3.6	42.9	×	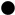	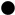	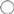	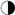	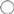	×
	Libelium Waspmote v15 [78]	2016	ATmega1281	8	14.75	128	8	4	15 modules available (e.g., Zigbee, LoRaWAN)		3.0	3.3–4.2	56.1	99.0	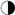	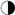	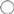	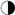	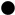	174.00
	Zolertia RE-Mote [79]	2016	CC2538SF53 ^g^	32	32	512	32	–	CC2538 ^g^ + CC1200	IEEE 802.15.4 (Zigbee)	3.3	3.3–16	66.0	4.3	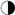	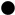	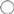	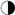	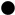	112.00
	WiSense WSN1120L [80]	2019	MSP430G2955	16	16	56	4	128	CC1120	sub-1 GHz narrowband	3.0	1.8–3.6	56.1	56.1	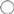	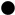	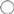	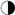	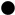	48.00
	OpenMote B [81]	2019	CC2538SF53 ^g^	32	32	512	32	–	CC2538 ^j^ + AT86RF215	IEEE 802.15.4/802.15.4g	3.3	2.0–3.6	42.9	4.3	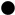	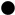	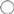	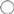	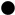	125.00
**Academia**	Kmote [82]	2007	MSP430F1611	16	8	8240 ^d^	10	16	CC2420	IEEE 802.15.4 (Zigbee)	3.3	2.3–6.0	4.9	22.1	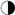	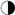	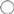	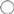	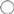	37.85
	Beasties [83]	2008	ATmega8L	8	4	8	33 ^e^	0.5	Radiometrix NiM2	433 MHz (proprietary)	5.0	7.0–20	77.5	40000	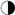	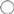	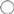	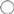	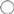	139.00
	INGA [84]	2012	ATmega1284P	8	4	128	16	4	AT86RF231	IEEE 802.15.4	3.3	×	61.7	×	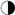	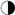	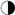	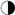	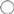	120.00
	Storm [85]	2014	ATSAM4LC8C	32	48	1536 ^f^	64	–	AT86RF233	IEEE 802.15.4	3.3	1.8–3.6	4.5	7.6	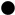	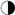	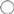	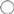	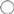	50.00
	Raju and Pratap [86]	2015	MSP430F5438	16	25	256	16	–	CC2520	IEEE 802.15.4 (Zigbee)	3.3	1.8–3.8	×	×	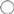	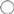	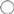	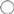	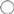	×
	Zeni et al. [72]	2015	ATmega328P	8	1	32	2	1	nRF24L01+	2.4 GHz (proprietary)	3.0	1.9–3.6	5.8	15	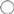	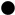	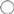	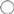	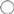	12.00
	panStamp NRG3 [87]	2016	CC430F5137	16	20	32	4	–	CC1101	433/868 MHz (proprietary)	3.3	2.0–3.6	46.2	8.3	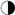	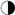	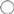	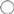	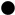	×
	EARNPIPE ^h^ [88]	2016	AT91SAM3X8E	32	84	512	100	–	×	IEEE 802.15.1 ^i^	3.3	7.0–12	×	×	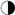	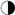	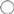	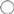	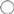	×
	uLoRa [89]	2017	STM32L051K8T6	32	32	64	8	2	DRF1272F	868 MHz (incl. LoRa)	3.3	×	34.7	1.2	×	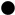	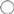	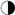	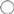	12.00
	Rusu and Dobra [90]	2017	STM32L443RC	32	80	256	64	–	AT86RF212B	IEEE 802.15.4 (ISA100)	3.3	×	×	×	×	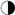	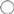	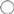	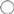	×
	Hamilton [58,91]	2017	ATSAMR21 ^g^	32	48	256	32	–	AT86RF233 ^g^	IEEE 802.15.4	3.0	×	3.2	19.5	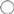	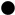	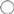	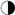	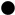	25.00
	Hazelnut [92]	2019	ATtiny85 ^j^	8	1	8	0.5	0.5	ESP8266 ^j^	IEEE 802.11 b/g/n	3.3	×	231	650	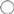	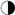	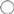	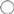	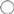	×
	Raposo et al. [54]	2019	MSP430F5229	16	25	128	8	–	Linear DC9003A-C	IEEE 802.15.4 (WirelessHART)	3.3	×	×	×	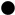	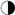	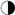	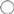	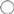	×
	Babusiak et al. [93]	2019	ATmega328P	8	1	32	2	1	nRF24L01+	2.4 GHz (proprietary)	3.0	1.5–3.6	10.5	22.2	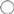	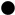	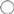	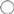	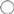	11.00
	MEGAN [94]	2020	ATmega324PA	8	8	32	2	1	Digi Xbee S2	IEEE 802.15.4 (Zigbee)	3.3	×	26.2	33.3	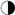	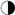	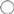	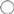	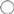	20.00
	ASN(x)	2021	ATmega1284P	8	4	128	16	4	Digi Xbee 3	IEEE 802.15.4 (Zigbee)	3.3	1.8–5.5	15.4	121.1	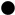	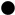	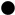	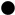	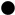	50.00
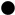 supported 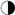 partly supported 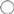 not supported – not available × no information available																				

^a^ 48 kB MCU + 1024 kB external; ^b^ 64 kB MCU + 180 kB external; ^c^ 128 kB MCU + 512 kB external; ^d^ 48 kB MCU + 8192 kB external; ^e^ 1 kB MCU + 32 kB external; ^f^ 512 kB MCU + 1024 kB external; ^g^ single-chip SoC including MCU & radio; ^h^ Arduino Due-based sensor node; ^i^ IEEE 802.15.1 version not stated; ^j^ ESP8266 is controlled by the ATtiny85 and can be used for resource-intense calculations.

**Table 2:** Overview of the current ASN(x) fault indicators.

Indicator		Category	Section
$\chi_{NT}$	node temperature monitor	artificial generic	Section 4.5.1
$\chi_{VS}$	supply voltage monitor	inherent component-specific	Section 4.5.2
$\chi_{BAT}$	battery voltage monitor	artificial generic	Section 4.5.3
$\chi_{ART}$	active runtime monitor	inherent component-specific	Section 4.5.4
$\chi_{RST}$	reset monitor	inherent component-specific	Section 4.5.5
$\chi_{IC}$	software incident counter	inherent common	Section 4.5.6
$\chi_{ADC}$	ADC self-check	artificial generic	Section 4.5.7
$\chi_{USART}$	USART self-check	artificial component-specific	Section 4.5.8

## Abbreviations

The following abbreviations are used in this manuscript:

ADC	analog-to-digital converter
ALU	arithmetic logic unit
AIS	artificial immune system
ASN(x)	AVR-based Sensor Node with Xbee radio
AVR	Alf and Vegard’s RISC
BLE	Bluetooth low energy
CH	cluster head
CMOS	complementary metal oxide semiconductor
CPS	cyber-physical system
CPU	central processing unit
DFS	dynamic frequency scaling
DSP	digital signal processor
DUT	device under test
DVS	dynamic voltage scaling
EEPROM	electrically erasable programmable read-only memory
ETB	embedded testbench
FPGA	field-programmable gate array
GPIO	general purpose input/output
HCI	hot carrier injection
I2C	inter-integrated circuit
IEEE	Institute of Electrical and Electronics Engineers
ISM	industrial, scientific and medical
ISP	in-system programmer
JTAG	Joint Test Action Group
LDO	low-dropout regulator
LED	light-emitting diode
LoRaWAN	long-range wide-area network
LPWAN	low-power wide-area network
MCU	microcontroller unit
MCUSR	MCU status register
MIPS	million instructions per second
MOSFET	metal-oxide-semiconductor field-effect transistor
NB-IoT	narrowband Internet of Things
NBTI	negative bias temperature instability
NTC	negative temperature coefficient
OS	operating system
OTR	outdoor relay node
OWI	one-wire interface
PCB	printed circuit board
RAM	random-access memory
RF	radio frequency
RISC	reduced instruction set computer
RSSI	received signal strength indicator
RTC	real-time clock
SDM	sequential dependency model
SK	sink node
SN	sensor node
SNR	signal-to-noise ratio
SoC	system-on-a-chip
SPI	serial peripheral interface
SQL	structured query language
SRAM	static random-access memory
SVM	support-vector machine
TDDB	time dependent dielectric breakdown
THT	through-hole technology
TWI	two-wire interface
USART	universal synchronous/asynchronous receiver-transmitter
ULP	ultra low power
USB	universal serial bus
WDT	watchdog timer
WSN	wireless sensor network
